# MiR319a-mediated salt stress response in poplar

**DOI:** 10.1093/hr/uhae157

**Published:** 2024-06-07

**Authors:** Yanxia Cheng, Qiao Wang, Linxi Yang, Quanzi Li, Xiaojing Yan

**Affiliations:** State Key Laboratory of Tree Genetics and Breeding, Chinese Academy of Forestry, Beijing 100091, China; College of Resources and Environment, Qingdao Agricultural University, Qingdao, Shandong 266109, China; State Key Laboratory of Tree Genetics and Breeding, Chinese Academy of Forestry, Beijing 100091, China; State Key Laboratory of Tree Genetics and Breeding, Chinese Academy of Forestry, Beijing 100091, China; State Key Laboratory of Tree Genetics and Breeding, Chinese Academy of Forestry, Beijing 100091, China

## Abstract

Maintenance of intracellular ion balance, especially Na^+^ and K^+^, plays an important role in plant responses to salt stress. Vessels in xylem are responsible for long-distance ion transport in vascular plants. Knowledge on the salt stress response in woody plants in limited. In this study, we identified miR319a as an important regulator in respond to salt stress in poplar. miR319a overexpression transgenic poplar showed a salt-tolerant phenotype, and cytological observation showed reduced cambium cell layers, wider xylem, increased number and lumen area of vessels and fibers, and thinner cell wall thickness in the transgenics. The miR319a-MIMIC plants, meanwhile, had opposite phenotypes, with narrower xylem, reduced number and lumen area of vessels and fibers cells, and increased wall thickness. In addition, overexpression of miR319a driven by the vessel-specific promoter significantly improved the salt tolerance compared with the fiber-specific promoter. The expression levels of *PagHKT1;2* and *PagSKOR1-b*, which encoded high-affinity K^+^ and Na^+^ transporters for Na^+^ efflux and K^+^ influx, respectively, were positively correlated with the vessel number and lumen area. These results suggest that miR319 not only promotes ion transport rates by increasing vessel number and lumen area and reducing cell wall thickness, but also regulates the concentrations of Na^+^ and K^+^ in the xylem by up-regulating *PagHKT1;2* and *PagSKOR1-b*. We demonstrate that miR319 may coordinate the response of poplar to salt stress through both mechanisms, enriching our understanding of the synergistic effects of the secondary xylem structure and long-distance ion transport balance in the salt tolerance of poplar.

## Introduction

Salt stress is one of the major environmental factors affecting plant growth and development and limiting high yield and good quality in plants [1–5]. The increased salt concentration in roots results in the reduced ability of plants to absorb water. Salt stress includes early-occurring osmotic stress, accumulating ionic stress [6], and some secondary stresses [7]. Osmotic stress is usually an instantaneous and rapid response caused by short-distance signals perceived by root cells [8]. Plants enact certain mechanisms to mitigate osmotic stress by reducing water loss while maximizing water uptake [9]. Once salt ions are taken up in large amounts by roots, they are transported over a long distance to the shoots, and finally accumulate in the leaves. The accumulation of Na^+^ ions in plant cells causes ionic stress, and it also destroys the absorption of plant cells to other non-salt ions, such as K^+^ [10]. Preserving the homeostasis of cellular ions stands as a vital adaptive characteristic in salt-tolerant plants when they are faced with an excess of ions, especially the imbalance of Na^+^ and K^+^ [11–13].

Due to their sessile properties, plants have developed a suitable mechanism in their long evolutionary history to respond to a high salinity environment, especially in the absorption and long-distance transport of Na^+^ and K^+^. Roots are the first organ to sense the change in salt ion concentration in soil [14]. In plant root cells, Na^+^ enters the cell via nonselective cation channels (NSCCs) or other membrane transporters including high-affinity K^+^ transporter 1 (HKT1) and Na^+^/H^+^ antiporter exchanger (NHX) Na^+^ transporters [15], whereas K^+^ enters the cell via K^+^ transporters high affinity K^+^ transporter (5HAK5 and Arabidopsis K^+^ transporter 1 (AKT1) [16, 17]. Through the sensory mechanism, Ca^2+^, reactive oxygen species (ROS) and hormone signaling cascades are activated [18–20] and a series of cellular detoxification signaling pathways, including the Na^+^ and K^+^ transporter signaling, are activated [11, 21]. HKT1 functions in the root-to-shoot Na^+^ partitioning, and it is the major player in plant salt tolerance by maintaining the Na^+^/K^+^ balance in tissues and cells through long-distance transport and distribution of Na^+^ [22]. Studies in *Arabidopsis thaliana*, *Oryza sativa*, *Triticum aestivum*, and *Solanum lycopersicum* show that the HKT1 protein mainly unloads Na^+^ from the xylem of roots through xylem parenchyma cells, and this uploading can prevent excess Na^+^ from being transported to the shoot that is caused by transpiration flow [23–27]. In blueberry, VcHKT1;1 promotes leaf Na^+^ exclusion by retrieving Na^+^ from xylem sap [28]. AtHKT1 in Arabidopsis is also involved in Na^+^ recycling from shoots to roots, probably by mediating Na^+^ loading into the phloem sap in shoots and unloading in roots [29]. AtHKT1 is expressed in the xylem and phloem of stamens and protects plant fertility by reducing Na^+^ content in stamen filaments [30]. HAK5 contributes to K^+^ entering into epidermis cells and AKT1 contributes to K^+^ retrieval from the xylem sap, whereas SKOR contributes to K^+^ translocation from xylem parenchyma to the xylem [16, 31]. Additionally, a member of the shaker family protein SKOR plays an important role in transporting K^+^ over long distances, especially in the translocation of K^+^ from roots to shoots [32]. At present, the long-distance ions transporting regulatory mechanism of salt stress response is mainly explored in annual herbaceous plants.

As perennial woody plants, trees have a unique secondary growth process in stems to maintain their stability and facilitate the transport of water and nutrients from roots to branches [33]. Under salty conditions, poplar trees display alterations of osmotic balance in the wood-forming tissues, with a decreased number of cambial cell layers, disorganized wood anatomical structures, and a change of the vacuoles into multiple smaller ones [34]. *Populus × canescens* trees exposed to salt have a decreased vessel diameter and an increased number of vessels, so that overall water conductivity is largely unaffected [35]. In addition, the wall thickness and hardness of poplar vessels are increased after salt stress, which can prevent vessel cell collapse under the osmotic pressure [36]. Anatomical changes in angiosperms after salt stress appear to be manifest primarily in vessels, whereas fiber and ray cells appear to be unchanged [34, 36]. Due to long growth cycles and unique secondary growth structures, trees should have evolved specific Na^+^/K^+^ long-distance transport mechanisms for acclimation and tolerance to salt stress, and this transport regulation mechanism may be different from that of annual herbaceous plants. However, there are few studies on this aspect in trees.

MicroRNAs (miRNAs) are 20–24 nucleotide non-coding RNAs that can target complementary sequences to negatively regulate gene expression [37, 38]. miR319 is one of the conserved miRNA families in herbaceous and woody plants [39, 40]. Its target *TEOSINTE BRANCHED*/*CYCLOIDEA*/*PROLIFERATING CELL FACTORS* (*TCP*) genes play important roles in plant growth and development, and response to biotic and abiotic stresses [41–43]. Overexpression of *MIR319* in Arabidopsis [44], tomato [45], rice [46], creeping bentgrass [43], and poplar [47] resulted in a significant alteration in leaf morphology and curvature. miR319 also plays a role in the formation of the vascular system. In Arabidopsis, miR319 overexpression results in a decrease in *TCP4* abundance and secondary cell wall thickness in the stem [48]. In *T. aestivum*, miR319 represses cell proliferation and cell expansion of culms [49]. In the seedling of *Populus tomentosa Carr.*, miR319a overexpression results in delayed secondary growth and decreased xylem production [50]. In summary, the mode of action of miR319 varies across different species and tissues. Although miR319-mediated changes in plant morphology and stress response have been well studied in herbaceous plants, there have been fewer published reports in woody plants, especially in terms of association between stem development and salt stress response.

In this study, we generated miR319a overexpression and suppression transgenic poplar plants. By analysing their growth phenotype and cytological phenotype in secondary xylem after salt stress treatment, we seek to answer a series of questions: Is miR319a involved in salt stress responses in woody plants? What impact may the salt stress have in the xylem development in poplar? And what is the crosstalk regulatory mechanism between miR319a-mediated secondary xylem development and salt stress response in poplar? Finally, we aim to explore the synergistic effects of the xylem structure and long-distance ion transport balance in poplar.

## Results

### miR319a is abundantly expressed in the stem of poplar and responds to salt stress

**Figure 1:**
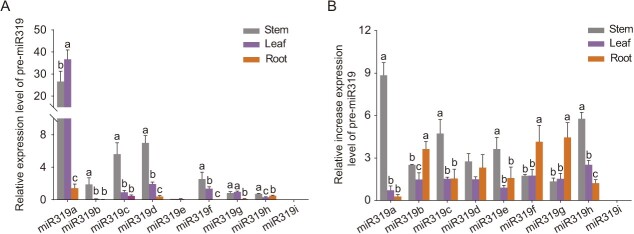
RT-qPCR analysis of miR319 abundance under control and saline conditions in poplar. **A** Abundance of miR319a-i in different tissues of poplar, including leaf, stem and root (shown in different colors). **B** The relative increase expression level of miR319a-i under 150 mM NaCl salt stress in different tissues, including leaf, stem, and root (shown in different colors). Statistical analysis of the relative expression level is shown (means ± SD, one-way analysis of variance (ANOVA)), and significant differences (*P* < 0.05) are indicated by different lowercase letters.

There are nine members of the miR319 family in *Populus alba × Populus glandulosa*, all of which have different stem-loop secondary structures with different pre-miRNA sequence and a similar 20 base-pair (bp) mature miRNA sequence (Fig. S1, see online supplementary material). They are divided into three groups based on the single base-pair variation of the 3′ terminus in the mature sequences. In group I, miR319a, −b, −c, and −d share identical mature miRNA sequences; in group II, miR319e, −f, −g, and −h share identical mature miRNA sequences; in group III, miR319i is unique in sequence (Fig. S1, see online supplementary material). Because their pre-miRNA sequences were different, we first examined the expression pattern of miR319 members in various tissues, including leaf, stem, and root. The RT-qPCR analysis detected a relatively high abundance of miR319a in the stem and leaf, and a low abundance in the root (Fig. 1A). To investigate the response of miR319 to salt stress in poplar, we treated poplar with 150 mM NaCl at about 7 days, and determined the miR319 accumulation level in different tissues after salt stress in leaf, stem, and root by RT-qPCR. The analysis showed that the relative increase expression level of miR319a in the stem was the most significant after NaCl treatment (Fig. 1B). These results indicated that the abundance of miR319a was relatively high in the stem and leaf of woody plants, and salt stress enhanced miR319a accumulation in the stem of poplar.

### miR319a positively regulates plant salt tolerance in poplar

To study the function of miR319a in the response to salt stress in poplar, we generated transgenic plants with enhanced and inhibited *MIR319a* expressions, respectively. Previously, we have generated the *MIR319a* overexpression transgenic poplar that harbored the stem-loop structure of *O. sativa MIR319a* (*Osa-MIR319a*) gene driven by the CaMV 35S promoter [47]. Two of the *OsaMIR319a* overexpression transgenic lines (*319-OE-1* and *319-OE-2*) were selected for subsequent studies. Target MIMICs are recognized for sequestering their target miRNAs, thereby influencing miRNA activity and stability [51]. Therefore, we transferred the *MIMIC319* generated form Arabidopsis *IPS1* (*INDUCED BY PHOSPHATE STARVATION1*) gene into poplar to obtain transgenic plants to reduce miR319a abundance in poplar. A total of 14 *miR319a-MIMIC* transgenic lines were generated (Fig. S2A, see online supplementary material), five of which were selected for further RT-qPCR. Compared with the wildtype (WT), the abundance of miR319a in these five transgenic lines was significantly reduced, and *319-MIM-22* and *319a-MIM-25* were chosen for further analysis (Fig. S2B, see online supplementary material).

**Figure 2:**
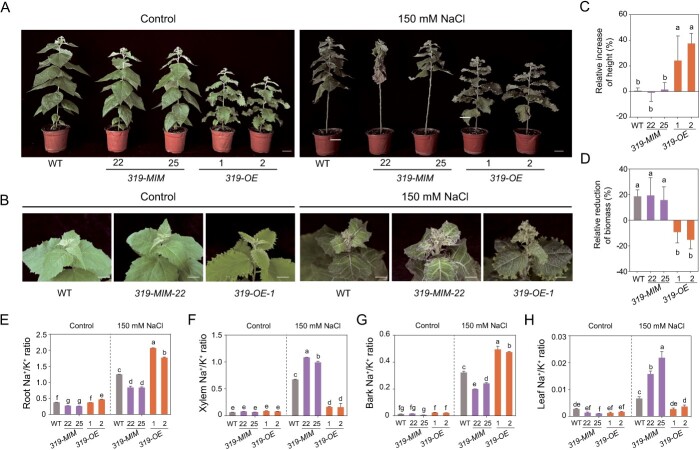
Phenotypic analysis of salt tolerance in wildtype (WT), *miR319a-MIMIC* and *miR319a-OE* transgenic poplars. **A** Under control and 150 mM NaCl salt treatment, appearance of 2-month-old WT, *miR319a-MIMIC* transgenic lines (*319-MIM-22* and *-25*) and *miR319a-OE* transgenic lines (*319-OE*-*1* and -*2*). Bar = 5 cm. **B** Under control and 150 mM NaCl salt treatment, top leaf phenotype of WT, *319-MIM-22*, and *319-OE-1* transgenic plants. Bar = 2 cm. **C**–**D** Relative height increase (**C**) and biomass reduction (**D**) in WT, *miR319a-MIMIC*, and *miR319a-OE* transgenic plants after 150 mM NaCl salt treatment. **E**–**H** Determination of Na^+^/K^+^ ratio in the root **(E)**, xylem **(F)**, bark **(G)**, and leaf **(H)** of WT, *miR319a-MIMIC*, and *miR319a-OE* transgenic plants under control and 150 mM NaCl. Statistical analyses are shown (means ± SD), one-way analysis of variance (ANOVA), and significant differences (*P* < 0.05) are indicated by different lowercase letters in **C**–**F**.

To investigate how miR319a affects the salt stress response in poplar, we observed the salt tolerance phenotype of 2-month-old WT, *miR319a-OE*, and *miR319a-MIMIC* transgenic plants maintained in nutrient soil. After treatment with 150 mM NaCl for 14 days, the leaves of *miR319a-MIMIC* transgenic plants (*319-MIM-22* and *-25*) exhibited more obvious wilting and chlorosis, compared to WT (Fig. 2A and B). While the growth of *miR319a-OE* transgenic plants (*319-OE-1* and *-2*) was more robust than that of WT (Fig. 2A and B), and the increases in height and biomass in *miR319a-OE* transgenic plants grown on 150 mM NaCl soil were significant (Fig. 2C and D). Maintaining the balance of Na^+^ and K^+^ is important for plant adaption to salt stress [52], especially in poplar stem xylem tissues that transport ions over long distances, so we detected the Na^+^ and K^+^ content in the different tissues from underground to aboveground of transgenic poplar. The Na^+^/K^+^ ratios in the root, xylem, bark, and leaf showed no significant differences between WT, *miR319a-OE*, and *miR319a-MIMIC* transgenic plants before 150 mM NaCl treatment (Fig. 2E–H). However, after salt treatment, in the root and bark, the Na^+^/K^+^ ratios of the *miR319a-MIMIC* transgenic plants were significantly reduced, while the Na^+^/K^+^ ratios in the *miR319a-OE* transgene were significantly increased compared with the WT. On the contrary, in the xylem and leaf, the Na^+^/K^+^ ratios of the *miR319a-MIMIC* transgenic plants were significantly increased, while the Na^+^/K^+^ ratios in the *miR319a-OE* transgene were significantly reduced compared with the WT (Fig. 2E–H). Taken together, these results suggested that *miR319a-MIMIC* transgenic plants accumulated more Na^+^ in xylem and leaf than WT plants, and *miR319a-OE* transgenic plants accumulated more Na^+^ in the root and bark. Therefore, we speculated that miR319a positively regulates salt tolerance in poplar by affecting the Na^+^ transport from root to shoot and Na^+^ efflux in xylem of stems.

The miR319a overexpression transgenic plants had heterologous overexpression of *Osa-MIR319a*. This Osa-miR319a had one base different with Pag-miR319a, the mature miR319a in *P. alba × P. glandulosa* (Fig. S3A, see online supplementary material). To further characterize the potential roles of poplar miR319a in responses to salt stress, we generated transgenic plant with the stem-loop structure of *Pag-MIR319a* gene under 35S promoter. A total of 4 *PagMIR319a* overexpression lines were generated, two of which (*Pag319–1* and *−4*) were selected for further analysis (Fig. S3B and C, see online supplementary material). We analysed the phenotypes of *miR319a-OE* and *PagmiR319a-OE* transgenic plants in response to salt stress. Under normal conditions, there was no obvious differences in growth between them (Fig. S3D, see online supplementary material). After 14 days of salt treatment, we observed that the leaf edge of *miR319a-OE* transgenic plants was turgid and bottle-green, whereas the leaf edge of WT and *PagmiR319a-OE* transgenic plants were flaccid and black, but the degree in WT plants was more serious (Fig. S3E, see online supplementary material). Both *miR319a-OE* and *PagmiR319a-OE* transgenic plants displayed a significant height increase compared with WT plants after salt stress (Fig. S3F, see online supplementary material). Consistently, the biomass of *319-OE-1* and *319-OE-2* transgenic plants after salt treatment was increased by 9.3% and 15.2%, respectively, while that of WT, *Pag319a-OE-1*, and *Pag319-OE-4* was decreased by 18.7%, 8.1%, and 7.6%, respectively (Fig. S3G, see online supplementary material). Compared with WT, Na^+^/K^+^ ratio in the shoot of *miR319a-OE* plants was decreased, while its level in *PagmiR319a-OE* plants was not changed significantly (Fig. S3H, see online supplementary material). The above results showed that *PagmiR319a-OE* transgenic plants also displayed a salt-tolerant phenotype, but the degree was not as strong as *miR319a-OE* transgenic plants. To further analyse the reasons for the different phenotypes between them, we identified the 36 TCP family members of miR319 target genes in *P. alba × P. glandulosa.* The RT-qPCR results showed that the expression level of *PagTCP3*, *4*, and *19* was down-regulated in both *miR319a-OE* and *PagmiR319a-OE* transgenic plants, but the degree of reduction was different between them. In addition, the expression level of *PagTCP2* was significantly down-regulated in *miR319a-OE* transgenic plants, but the change was not significant in *PagmiR319a-OE* transgenic plants, while the expression level of *PagTCP20* was significantly down-regulated in *PagmiR319a-OE* transgenic plants, but the change was not significant in *miR319a-OE* transgenic plants (Fig. S3I, see online supplementary material). These results proved that the PagmiR319a also positively regulates salt tolerance in poplar, and its phenotype may not be completely consistent with that of *miR319a-OE* transgenic plants due to the different expression levels of target *TCP* genes.

### miR319a regulates the genes related to salt stress response and xylem cell wall development

Xylem is the main tissue for transporting Na^+^ and K^+^, especially in the secondary xylem unique to woody plants. Therefore, to further explore the molecular mechanism of miR319a in regulating salt stress, we conducted high throughput transcriptome sequencing (RNA-seq) on the differentiating xylem tissue in WT, *miR319a-MIMIC*, and *miR319a-OE* transgenic plants. The differentially expressed genes (DEGs) were screened with a log_2_ fold change range of <−2 and >2 with a *P*-value <0.05. *miR319a-MIMIC* transgenic plants had 67 DEGs, of which 46 were up-regulated (Fig. S4A, Table S1, see online supplementary material) and 21 were down-regulated (Fig. S4A, Table S1, see online supplementary material). While in *miR319a-OE* transgenic plants, a total of 492 DEGs were obtained, of which 462 (Fig. S4B, Table S2, see online supplementary material) were up-regulated and 30 were down-regulated (Fig. S4B, Table S2, see online supplementary material). GO ontology (GO) analysis showed that most of the DEGs in *miR319a-MIMIC* and *miR319a-OE* transgenic plants could be classified into two categories: one was related to the plant salt stress response process, and the other was related to xylem cell wall development (Fig. S4C, see online supplementary material). RNA-Seq analysis showed that miR319a mainly affects the expression of the genes related to xylem cell wall development and response to salt stress.

**Figure 3:**
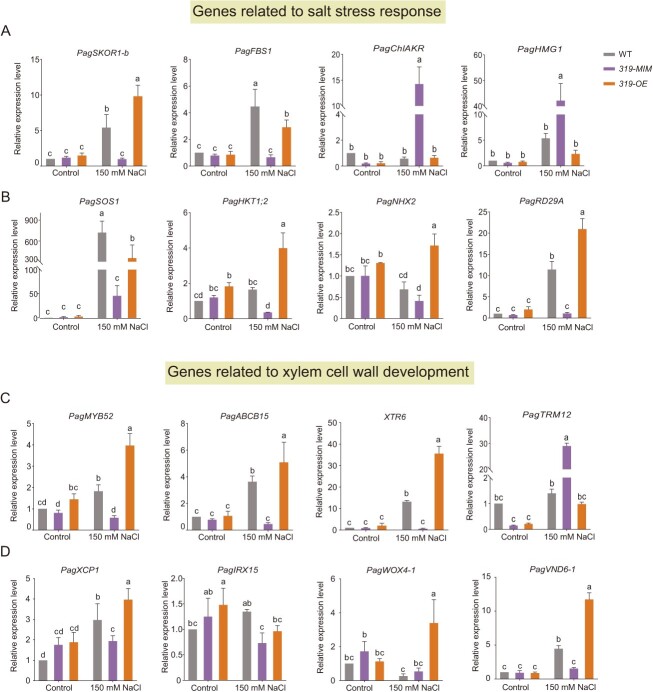
RT-qPCR analysis of salt stress-related genes and xylem development-related genes in the differentiating xylem of wildtype (WT), *miR319a-MIMIC* (*319-MIM-22*), and *miR319a-OE* (*319-OE-1*) transgenic poplars under control and 150 mM NaCl treatment. **A** Expression levels of the genes related to salt stress response. **B** Expression levels of the reported genes related to salt stress response. **C** Expression levels of the genes related to xylem cell wall development. **D** Expression levels of the reported genes related to xylem cell wall development. Statistical analyses of the relative expression level are shown (means ± SD), one-way analysis of variance (ANOVA), and significant differences (*P* < 0.05) are indicated by different lowercase letters).

To verify the RNA-Seq results and detect the expression level of the two types of genes under salt stress, we selected some genes in the sequencing results and performed RT-qPCR. In the category related to salt stress response, the most important one is *PagSKOR1-b* (GO:0071805), a protein responsible for long-distance transport of K^+^. The expression level of this gene was increased significantly after salt stress, with the highest expression level in *miR319a-OE* transgenic plants and the lowest expression level in *miR319a-MIMIC* transgenic plants (Fig. 3A). The expression levels of abiotic stress response factor genes *PagFBS1* (GO:0009651), *PagChlAKR* (GO:0009651), and *PagHMG1* (GO:0098739) were significantly changed after salt stress (Fig. 3A). At the same time, we also detected the genes that have been reported to function in salt stress response. As shown in Fig. 3B, compared with the WT, the expression levels of *PagHKT1;2*, *PagSOS1*, and *PagNHX2*, which regulate ion transport, were decreased in *miR319a-MIMIC* and increased in *miR319a-OE* transgenic plants*.* The *PagRD29A* gene related to abiotic stress was also down-regulated in *miR319a-MIMIC* and up-regulated in *miR319a-OE* transgenic plants (Fig. 3B). In another group of genes related to xylem cell wall development, the transcription factor gene *PagMYB52* (GO:0009664), which regulates secondary cell wall biosynthesis, was up-regulated in *miR319a-OE* and down-regulated in *miR319a-MIMIC* after salt stress (Fig. 3C). *PagABCB15* (GO:0098656), encoding a key regulatory factor involved in the formation of xylem vessels, and *PagXTR6* (GO:0009664), encoding a key enzyme for the synthesis of cell wall components, were up-regulated in *miR319a-OE* and down-regulated in *miR319a-MIMIC* after salt stress (Fig. 3C). *PagTRM12* (GO:0003674), which was related to cell division and differentiation, was up-regulated in *miR319a-MIMIC* and down-regulated in *miR319a-OE* after salt stress (Fig. 3C). In addition, the expression levels of regulatory genes involved in the formation of poplar xylem vessels (*PagXCP1*), secondary cell wall deposition (*PagIRX15*), secondary cell wall formation (*PagVND6*), and cambial cell activity (*PagWOX4*) were increased in *miR319a-OE* and decreased in *miR319a-MIMIC* transgenic plants (Fig. 3D). The above analysis results showed that miR319a mainly regulated the expression of salt stress response and xylem cell wall development genes in poplar**.**

**Figure 4:**
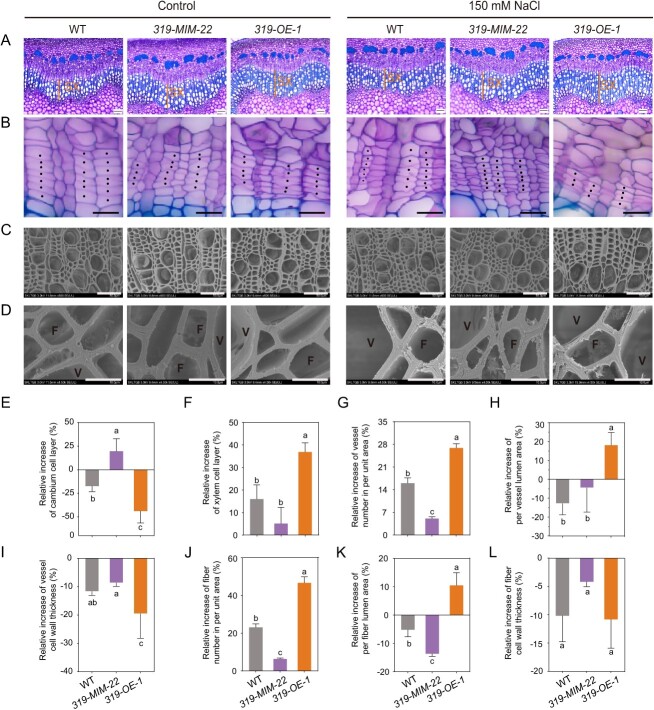
Secondary xylem development-related phenotypic analysis in wildtype (WT), *miR319a-MIMIC*, and *miR319a-OE* transgenic poplars. **A**–**B** Stem cross-sections of the 10th internode in WT, *miR319a-MIMIC* (*319-MIM-22*), and *miR319a-OE* (*319-OE-1*) transgenic plants under control and 150 mM NaCl salt treatment. Bar = 100 μm. SX, secondary xylem (**A**); Bar = 30 μm. Solid black dots indicate the cambium cells (**B**). **C**–**D** SEM images of stem cross-sections of the 10th internode in WT, *miR319a-MIMIC* (*319-MIM-22*), and *miR319a-OE* (*319-OE-1*) transgenic plants under control and 150 mM NaCl salt treatment. Bar = 50 μm (**C**) and 10 μm (**D**). V, vessel; F, fiber. **E**–**L** Relative increase of cambium cell layers (**E**), xylem cell layers (**F**), vessel number in an area of 300 **×** 150 mm^2^ (**G**), vessel lumen area (**H**), vessel cell wall thickness (**I**), fiber cell number (per unit area) (**J**), fiber lumen area (**K**), and fiber cell wall thickness (**L**) of the 10th internode in 2-month-old WT, *miR319a-MIMIC*, and *miR319a-OE* transgenic plants after salt treatment. Values are shown by means ± SD, and statistical analyses were performed using one-way analysis of variance (ANOVA), and the significant differences (*P* < 0.05) are indicated by different lowercase letters in **E**–**L**.

Among these DEGs, *PagHKT1* and *PagSKOR* encoded key proteins in regulating long-distance transport of Na^+^ and K^+^ from root to shoot [53, 54]. We constructed the phylogenetic evolution tree for HKT and SKOR proteins, including two HKT1s (PagHKT1;1 and HKT1;2) (Fig. S5A, see online supplementary material) and six SKORs (PagSKOR1-a, SKOR1-b, SKOR2-a, SKOR2-b, SKOR3-a, and SKOR2-b), identified in *P. alba × P. glandulosa* (Fig. S5B, see online supplementary material). Only two of them, *PagHKT1;2* and *PagSKOR1-b*, had significant changes in their gene expression levels (Fig. 3A and B).

### miR319a regulates the secondary xylem development in poplar stems after salt stress

RNA-Seq analysis showed that in addition to affecting the expression of the genes related to salt stress and ion homeostasis, miR319a also regulated the developmental process of xylem cell wall formation. To further explore the mechanism of miR319a in response to salt stress, we observed and analysed the secondary growth structure of the stems of poplar. Stem cross-sections of the 10th internode undergoing secondary growth were analysed in 2-month-old WT and transgenic plants under 150 mM NaCl treatment. After salt treatment, it was found that compared with control (without salt treatment), the number of cambium cell layers in *miR319a-MIMIC* transgenic plants were increased by 19.6% (Fig. 4B and E), and the layers of secondary xylem were increased by 5.1% (Fig. 4A and F). However, in *miR319a-OE* transgenic plants, the number of cambium cell layers was decreased by 43.8% (Fig. 4B and E), and the number of secondary xylem layers was increased by 36.9% (Fig. 4A and F). Vessels are the main channels for transporting water and inorganic salt ions in plants. Therefore, we further examined the xylem vessels in the stem cross-sections of the 10th internode in WT and transgenic plants by scanning electron microscopy (SEM). Compared with control, *miR319a-MIMIC* transgenic plants under salt stress had a relatively increased number of vessels (per unit area) (9.1%) (Fig. 4C and G), a relatively decreased vessel lumen area (4.4%) (Fig. 4D and H), and a relatively reduced vessel cell wall thickness (8.6%) in Fig. 4D and I. While *miR319a-OE* transgenic plants under salt stress displayed a higher extent of alteration compared to *miR319a-MIMIC* plants, with the relative increase of 26.9% in vessel number (Fig. 4C and G), the relative increase of 18.2% in vessel lumen area (Fig. 4D and H), and the relative reduction of 19.5% in vessel cell wall thickness (Fig. 4D and I). We also observed that fiber cells had the same changes as vessels after salt treatment. In *miR319a-MIMIC* and *miR319a-OE* transgenic plants, the relative increase in the number of xylem fibers (per unit area) was 6.3% and 46.6%, respectively (Fig. 4C and J), the relative increase of the xylem fiber lumen area was −13.7% and 10.5%, respectively (Fig. 4D and K), and the relative reduction of xylem fiber cell wall thickness was 4.17% and 10.8%, respectively (Fig. 4D and L).

The above cell biology and statistical results showed that after salt stress the number of cambium layers in poplar stems was decreased, the secondary xylem became wider, the number of vessels and fiber cells (per unit area) was increased and their secondary walls became thinner. Moreover, these changes were positively correlated with salt tolerance. The relative change value was the most obvious in *miR319a-OE* transgenic plants, followed by WT, and the change in *miR319a-MIMIC* transgenic plants was the weakest. These results also implied that miR319a plays an important role in the development of secondary xylem cells after salt stress. miR319a may regulate the response of poplar to salt stress by reducing the thickness of secondary cell walls and increasing the lumen area of vessels and fibers.

### miR319a improves the salt tolerance of poplar by affecting secondary xylem development and *PagHKT1;2*/*PagSKOR1-b* gene expression

The above results indicated that the miR319a-mediated salt stress response in poplar may be related to the secondary xylem development. At the same time, we also observed the salt stress phenotypes of WT and *miR319a-OE* transgenic seedlings grown on the medium. After 150 mM NaCl treatments for 20 days, WT and *miR319a-OE* transgenic plants exhibited similar salt-sensitive phenotypes. Because there is no significant secondary growth process in the stems of smaller poplar tissue culture seedlings, this result suggested that the secondary growth of poplar may mediate the response to salt stress, providing more solid evidence for the correlation between miR319a-mediated salt stress response and the stem secondary growth process of trees (Fig. S6, see online supplementary material).

In order to further explore the relationship between response of miR319a to salt stress and the regulation of miR319a on the development of vessels and fiber cells in secondary xylem, we used the vessel- and fiber cell-specific promoters (*PdXCP1p* and *PdDUF579–9p*) [55] to drive *MIR319a* overexpression, respectively, and performed genetic transformation. A total of seven *PdXCP1p-35Smini-miR319a* (*V-miR319a*) transgenic lines were generated, two of which (*V-319-13* and *V-319-18*) with the highest expression level were selected for the next research (Fig. S7A and B, see online supplementary material). Moreover, a total of nine *PdDUF579–9p-35Smini-miR319a* (*F-miR319a*) transgenic lines were generated, two of which (*F-319-9* and *F-319-15*) with the highest expression level were selected for further analysis (Fig. S7C and D, see online supplementary material).

**Figure 5:**
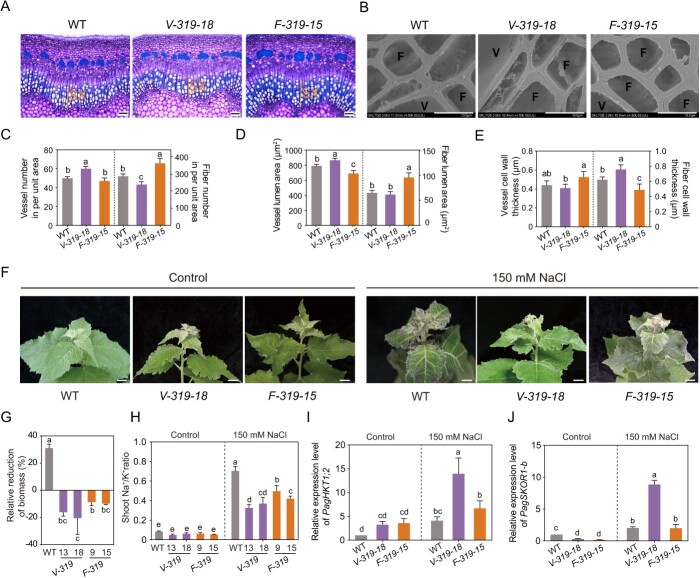
Phenotypic analysis of secondary xylem and salt tolerance of wildtype (WT) *V-miR319a* and *F-miR319a* transgenic poplars. **A** Stem cross-sections of the 10th internode in WT, *V-miR319a* (*V-319-18*), and *F-miR319a* (*F-319-15*) transgenic plants. Bar = 100 μm. SX, secondary xylem. **B** Scanning electron microscopy (SEM) images of stem crosss-section of the 10th internode in WT, *V-miR319a* (*V-319-18*), and *F-miR319a* (*F-319-15*) transgenic plants. Bar = 10 μm. V, vessel; F, fiber. **C**–**E** Statistical measurement of vessel and fiber cell number in an area of 300 **×** 150 mm^2^ (**C**), average lumen area of per vessel and fiber (**D**), and wall thickness of vessels and fiber cells (**E**) of the 10th internodes in 2-month-old WT, *V-miR319a* (*V-319-18*) and *F-miR319a* (*F-319-15*) transgenic plants. **F** Under control and 150 mM NaCl salt treatment, top leaf phenotype of WT, *V-miR319a* (*V-319-18*), and *F-miR319a* (*F-319-15*) transgenic plants. Bar = 2 cm. (**G**–**H**) Statisticl measurement of relative reduction of biomass (**G**) and shoot Na^+^/K^+^ ratio (**H**) in WT, *V-miR319a* (*V-319-13* and *− 18*), and *F-miR319a* (*F-319-9* and *− 15*) transgenic plants after 150 mM NaCl salt treatment. **I**–**J** RT-qPCR analysis of *PagHKT1;2* (**I**) and *PagSKOR1-b* (**J**) in the differentiating xylem of WT, *V-miR319a* (*V-319-18*), and *F-miR319a* (*F-319-15*) transgenic plants under control and 150 mM NaCl treatment. The statistical analyses are shown (means ± SD, one-way analysis of variance (ANOVA), significant differences (*P* < 0.05) are indicated by different lowercase letters) in **C**–**E** and **G**–**J**.

We cross-sectioned the poplar stems of the 10th internode and examined the development of secondary xylem. Compared with the WT, the *V-miR319a* transgenic plants displayed increases in vessel number (per unit area) (20.3%) (Fig. 5A and C; Fig. S8A, see online supplementary material) and vessel lumen area (9.5%) (Fig. 5B and D), and a reduction in vessel cell wall thickness (6.9%) (Fig. 5B and E). While *F-miR319a* transgenic plants exhibited a larger extent in the changes, including increases of 26.5% (Fig. 5A and C; Fig. S8A, see online supplementary material) in fiber cell number (per unit area) and 47.3% (Fig. 5B and D) in fiber lumen area, and a reduction of 21.5% in fiber cell wall thickness (Fig. 5B and E). We also found that compared with the WT, the fiber cell walls of *V-miR319a* transgenic plants and the vessel cell walls of *F-miR319a* transgenic plants were thicker (Fig. 5E). Both the vessel- and fiber-specific promoters are originally discovered in the xylem of Arabidopsis. *XCP1* is specifically expressed in vessel elements [56, 57], and DUF579–9 is specifically expressed in fibers and ray cells [58]. Although studies have proven the specificity of them in poplar, due to the complex structure of poplar xylem and the high degree of gene function differentiation, both may also have a lower expression level in other xylem cells, resulting in the changes of fiber cell wall thickness in *V-miR319a* plants and the changes of affected vessel cell wall thickness in *F-miR319a* plants. In addition, our research found that miR319 could negatively regulate the thickness of cell walls. In *V-319-18* transgenic plants, the vessel cell walls become thinner, but the poplar trees show a normal growth phenotype. At the same time, the fiber cell walls become thicker. The *F-319-15* transgenic plants showed the opposite state. It is speculated that this phenomenon may be because a coordinated regulation mechanism spontaneously formed by poplar to maintain the normal upright growth of their stems. These *V-miR319a* and *F-miR319a* transgenic plants were subjected to 150 mM NaCl treatments for 14 days, followed by phenotypic observations. After salt treatment, the leaves of WT plants exhibited obvious wilting and chlorosis, while the leaves of *F-miR319a* transgenic plants only displayed obvious chlorosis, and *V-miR319a* transgenic leaves did not show significant phenotypes of wilting and chlorosis (Fig. 5F; Fig. S8B, see online supplementary material). Compared to the biomass production of 31.0% in WT, *V-miR319a* and *F-miR319a* transgenic plants had biomass increases of 18.3% and 9.2%, respectively (Fig. 5G). Consistently, the plant height of *V-miR319a* and *F-miR319a* plants was significantly higher than that of the WT (Fig. S8C, see online supplementary material). The same tolerance trend was also reflected on the Na^+^ content in shoots (Fig. S8D, see online supplementary material). Compared with WT, the Na^+^/K^+^ ratio was reduced by 50.7% in *V-miR319a* transgenic plants and 35.1% in *F-miR319a* transgenic plants (Fig. 5H).

We further detected the expression levels of *PagHKT1;2* and *SKOR1-b* in the differentiating xylem of *V-miR319a* and *F-miR319a* transgenic plants. The RT-qPCR analysis showed that compared with WT, both *PagHKT1;2* and *PagSKOR1-b* were up-regulated in *V-miR319a* transgenic plants, and their expression levels did not change significantly in *F-miR319a* transgenic plants (Fig. 5I and J). The specific up-regulation of *PagHKT1;2* and *PagSKOR1-b* and the higher salt tolerance in *V-miR319a* than in *F-miR319a* suggest that miR319a can improve the salt tolerance of poplar by increasing vessel number and lumen area, and this phenomenon is positively correlated with the expression level of *PagHKT1;2* and *PagSKOR1-b* genes.

## Discussion

**Figure 6:**
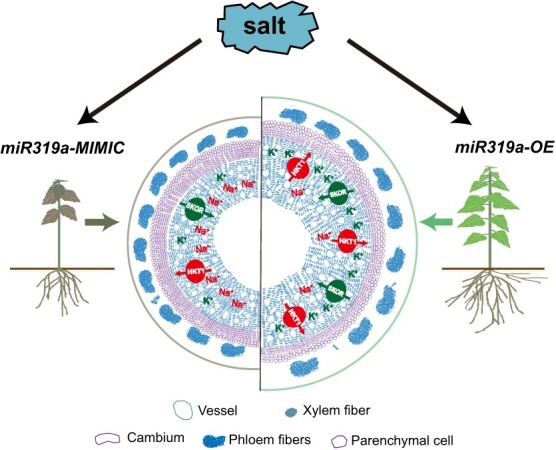
A proposed model of the miR319a-mediated enhancement salt tolerance in poplar.

miR319 is a highly conserved small RNA in plants and is involved in multiple biological processes, including plant growth and development and response to stress in herbaceous plants [51]. However, it has been less studied in woody plants. In this study, we created transgenic poplar plants with overexpression and silencing of miR319a, and further phenotypic characterizations and analysis showed that miR319a engages in crosstalk with stem secondary growth to regulate the plant response to salt stress. In *miR319a-OE* transgenic plants, on the one hand, the number of cambial cell layers in the stem were reduced, the xylem became wider, the number and lumen area of both vessels and fiber cells were increased, and the cell wall became thinner. On the other hand, the expression levels of the *PagHKT1* gene, which regulates Na^+^ efflux, and the *PagSKOR* gene, which regulates K^+^ influx, were increased significantly. Due to the changes in the cell structure of xylem, which is related to long-distance ion transport and the alterations in the expression levels of ion transport proteins mentioned above, there is a phenomenon of reduced Na^+^ content and increased K^+^ content in the xylem. As a result, the *miR319a-OE* transgenic plants exhibit a salt-tolerant phenotype (Fig. 6). While *miR319a-MIMIC* transgenic plants exhibited opposite phenotypes, with the number of cambial cell layer increased, xylem narrower, number and lumen area of both vessels and fiber cells decreased, and wall thickness increased. In addition, the expression levels of *PagHKT1;2* and *PagSKOR1-b* in *miR319-MIMIC* were reduced. Exactly due to these changes, the balance of Na^+^ and K^+^ in the xylem is disrupted, resulting in an increase of Na^+^ content and a decrease in K^+^ content. Therefore, the *miR319-MIMIC* plants exhibit a salt-sensitive phenotype (Fig. 6).

The expression level of miR319 is regulated by environmental stimuli, suggesting its involvement in plant stress response. Under salt stress, miR319 expression level was found to be up-regulated in Arabidopsis [59], wheat [60], and switchgrass [51], but down-regulated in maize [61] and *Solanum linnaeanum* [62]. However, specific experimental data are lacking, and the functional mechanism remains elusive in poplar. In this study, we found that salt stress enhanced miR319a accumulation in the xylem of poplar. Overexpression of both *OsamiR319a* and *PagmiR319a* could enhance the salt tolerance of poplar trees, but the phenotype of *OsamiR319a-OE* is more significant. This may be due to the one base difference between the two miRNAs, and their downstream target genes are not identical. TCP is the most comprehensive target transcription factor of miR319 reported in current research. Studies have shown that miR319a increases leaf serration and trichome density in poplar through *TCP4* [63] and *TCP19* [64], and regulates secondary growth of poplar by *TCP20* [50]. Through transcriptome profiling and GO analysis, we identified a series of genes related to salt stress, ion transport and cell wall formation, as well as some TCP genes. *PagTCP2*, *3*, *4*, *19*, and *20* were down-regulated in *miR319a-OE* or *PagmiR319a-OE* transgenic plants, but the degree of reduction was different between them. Because the direct target gene of miR319 is often TCP, to analyse the function of miR319 more accurately and comprehensively in the salt stress response in poplar, in-depth research and analysis of these TCP transcription factors are needed in the future. In addition, miR319a can positively regulate the expression of *PagHKT1;2*, *PagSKOR1-b*, and some genes related to salt stress and xylem development. Interestingly, these genes had lower expression levels under normal conditions, and their expression levels increased after salt stress treatment, while were extremely higher in *miR319a-OE* transgenic plants than in WT plants after salt stress. This phenomenon may be because the expression of *MiR319a* is also induced by salt stress. These results imply that salt stress and miR319 coordinately regulate the expression of perception and signaling genes.

Herbaceous plants are typically annual or perennial, with shorter stature, primarily growing through leaves and the basal parts below the ground. Woody plants generally have a long lifespan and can survive for many years. They are usually relatively tall, with the trunk as the main body, increasing the volume and circumference through secondary growth of the stem, and having a hard and special stem structure [65]. Due to the above-mentioned differences, woody plants and herbaceous plants may exhibit distinct physiological characteristics and regulatory mechanisms when facing salt stress. The Na^+^ and K^+^ absorbed by the roots can be transported to the aboveground parts through the plant vascular tissue system under the action of transpiration pressure, through the stems, and finally distributed to the aboveground leaves [14]. Woody plants specifically express some genes related to xylem secondary growth and lignin biosynthesis to regulate ions long-distance transport and maintain the stability of their cell walls, thereby maintaining the balance and homeostasis of ions in plants. Studies have shown that salt stress can affect the xylem structure and chemical components of trees. However, the extent of these changes in xylem structure may be influenced by within-tree species differences. For example, in *Populus canescens* salt stress causes a decrease in vessel diameter and an increase in vessel numbers [36], while salt-sensitive species respond to salt stress more quickly and significantly than salt-tolerant species such as *Populus euphratica*, which exhibit only minor anatomical changes even under severe salinity [66]. Therefore, due to long growth cycles and unique secondary growth structures, trees have evolved specific ions long-distance transport mechanisms for acclimation and tolerance to salt stress, and this response mechanism may not be the same in different trees. In this study, we used hybrid poplar (*P. alba × P. glandulosa*) as the research object and found that salt stress can cause a reduction of the number of cambium cell layers and an increase of the xylem lumen area. Furthermore, vessel- and fiber-specific expression of miR319a in poplar can enhance salt tolerance, but the phenotype of vessel-specific expression is more significant in *P. alba × P. glandulosa*. The specific up-regulation of *PagHKT1;2* and *PagSKOR1-b* and the higher salt tolerance in *V-miR319a* than in *F-miR319a* suggest that miR319a can improve the salt tolerance of poplar by increasing vessel number and lumen area, and this phenomenon is positively correlated with the expression level of *PagHKT1;2* and *PagSKOR1-b* genes in *V-miR319a* transgenic plants. However, in *F-miR319a* transgenic plants the lumen area of vessel cells was reduced, but it also showed slightly stronger salt tolerance phenotype than the wild type. It is speculated that this may be due to the enlargement of the fiber cell lumen area that ions are translocated from the vessel into the fiber cell, causing it to assume part of the function of transporting ions. These results provide genetic resources and the theoretical basis for using genetic engineering technology to create a new salt-tolerant poplar germplasm.

In conclusion, we characterized the functional mechanism of miR319 in the coordinated regulation of secondary xylem development and salt stress response in poplar. The *miR319a-OE* plants showed a salt-tolerant phenotype, with reduced cambial cells, wider xylem, increased number and lumen area of vessels and fibers, and thinner cell wall thickness. The *miR319a-MIMIC* plants showed a salt-sensitive phenotype, with an opposite phenotype as *miR319a-OE* plants. In addition, through phenotypic analysis of transgenic plants specifically expressing miR319 in vessels and fiber cells, it was found miR319a can improve the salt tolerance of poplar by increasing the number and lumen area of vessels and reducing the thickness of the cell wall. At the same time, the expression levels of *PagHKT1;2* and *PagSKOR1-b*, which were responsible for Na^+^ efflux and K^+^ influx, are positively correlated with the number and lumen area of the vessels. Overall, our results show that miR319 plays an important role in woody plants’ response to salt stress by regulating xylem vessel number, lumen area, cell wall thickness and ion transport gene expression levels. The structural alteration in the vessel plays a more important role than in fiber cells. Whether the alteration of wall thickness in the vessel is involved in response to salt stress needs further investigation.

## Materials and methods

### Plant materials and growth conditions

Hybrid poplar (*P. alba × P. glandulosa* ‘84 K’) was used for genetic transformation, gene cloning, and expression analysis. Poplar seedlings were asexually propagated in Murashige and Skoog (MS) nutrient medium (1/2 MS base medium +0.2 mg/L NAA + 0.05 mg/L IBA pH = 5.8–5.9) and maintained in the growth chamber at 25°C under a 16 h light/8 h dark cycle photoperiod (light intensity was 200 μmol/m^2^/s) conditions. The tissue-cultured seedings at nearly one month old were transplanted into 8 × 8 cm plastic pots containing 3:1 (m/m) mixtures of soil:perlite and grown in the growth chamber.

### Plasmid construction and plant transformation

The stem-loop of *OsamiR319a* was cloned from *pZH01:OsamiR319a-OE* plasmid, which was obtained from Prof. Dayong Li (Beijing Academy of Agriculture and Forestry Sciences). The *miR319a-MIMIC* plasmid was obtained from Prof. Wanjun Zhang (China Agricultural University). The stem-loop of *OsamiR319a* was fused with the vectors of *pCAMBIA2300:PdXCP1p-35S mini-GUS* and *pCAMBIA2300:PdDUF579–9p-35S mini-GUS* [55] by means of homologous recombination. The stem-loop of *PagmiR319a* was cloned from *P. alba × P. glandulosa* and then was fused with the *OsamiR319a-OE* vector and obtained *PagmiR319a-OE* by means of homologous recombination. These vectors were transformed into *P. alba × P. glandulosa* via the agrobacterium mediation leaf disc method. For transformation, the third to fifth leaves of 3–4 weeks wide type seedlings were snipped off petiole and cut main vein with scissors and scalpel. After soaking in the solution of *Agrobacterium* for 12–15 min, the injured leaves were transferred to a differentiation medium (MS base medium +0.5 mg/L 6-BA +0.05 mg/L NAA, pH = 5.8–5.9) at 23–25°C for 3–4 days under dark culture conditions, then leaves were transferred into a selection medium (MS base medium +0.5 mg/L 6-BA +0.05 mg/L NAA + 200 mg/L Timentin +50 mg/L Kanamycin, pH = 5.8–5.9) at 23–25°C under 16 h/8 h light/dark photoperiod conditions. The infected leaves were transferred to a new selective culture every 10 days until the buds grew to 1 cm. The buds were transplanted into 1/2 MS rooting medium (1/2MS base medium +0.2 mg/L NAA + 0.05 mg/L IBA + 200 mg/L Timentin +50 mg/L Kanamycin, pH = 5.8–5.9). Finally, the transgenic plants were used for further research after many instances of asexual reproduction. Genomic DNA was extracted as described previously [67] for PCR analysis on a T100TM Thermal Cycler BIO-RAD (Hercules, California, USA).

### Determination of miRNA abundance

Total RNA was extracted from different tissues. The differentiating xylem was scraped from the xylem side of the stem 6th internode to 14th internode in 2-month-old WT and transgenic plants using the standard CTAB method [68]. Double-stranded cDNA was synthesized using the miRNA cDNA synthesis kit with Poly (A) polymerase Tailing abm® (Vancouver, Canada). The qPCR reactions were run on Roche LightCycler 480 II detection machine using the Green Premix Ex Taq II Takara (Dalian, China) with the primers TTGGACTGAAGGGTGCTCCC and Universal 3’miRNA Reversal Primer abm® (Vancouver, Canada). A nuclear small RNA 5.8 s was used as internal control to normalize gene expression level [69]. △△Ct method was used for relative expression level analysis, and 2^-△△Ct(average)^ was used for normalizing the data [70]. The primers used are listed in Table S3 (see online supplementary material).

### Histological observations

Stem cross-sections of 30 μm were prepared from the 10th internode in 2-month-old WT and transgenic plants using a vibratome Leica VT1000s (Nussloch, Germany). Sections were stained in 0.05% toluidine blue O for 2 min and observed under an OLYMPUS BX51 microscope (Tokyo, Japan). All images calculated the layers of cambium and xylem using Image J software.

### Scanning electron microscopy (SEM) analysis

Area and cell wall thickness of vessels and fiber cell were measured in the SEM images. Firstly, stem cross-sections of the 10th internode were cut to a length of approximately 2 mm and immersed in 4% glutaraldehyde fixative Coolaber (Beijing, China) at 4°C for 4 h. The tissues were dehydrated under gradient alcohol (30%, 50%, 70%, 80%, 90%, 95%, and 100%) 15–20 min and dried by carbon dioxide critical points Leica EM CPD300 (Nussloch, Germany). Stem segments were glued to the stage and observed under a SEM Regulus 8230 (Tokyo, Japan). Per vessel and fiber of area and cell wall thickness were measured and counted using image J.

### Salt stress assays

For salt stress assays, 2-month-old WT and transgenic plants grown in soil were treated with salt-free water and 150 mM NaCl salt solution for 14 days, respectively. At the same time, the growth changes of poplars were observed and recorded with the camera Canon EOS70D (Tokyo, Japan), and the heights of poplars were measured with tapeline. Differentiating xylem was quickly scraped and frozen in liquid nitrogen to extract RNA for RT-qPCR analysis. We collected the root, xylem, bark, leaf and shoots (stem and apical) of each line after non-saline and saline treatment and measured the dry weight of each line and Na^+^ and K^+^ content.

### Measurement of Na^+^ and K^+^ concentration

The shoots of WT and transgenic plants were dried in an oven at 60°C, and milled to a fine powder by a vibration mill Ant group AM100s (Beijing, China). The constant-weight materials were digested with HNO_3_-H_2_O_2_ in a microwave-accelerated reaction system CEM (Charlotte, North Carolina, USA) until the solution and sediment became clear and milky. The Na^+^ and K^+^ concentrations were determined by ICP-MS Agilent 7700x (Santa Clara, California, USA).

### RNA-Seq analysis and RT-qPCR

Total RNA of the differentiating xylem in WT, *miR319a-MIMIC-22*, *miR319a-MIMIC-25*, *miR319a-OE-1*, and *miR319a-OE-2* transgenic plants was extracted using the CTAB method [68]. RNA-Seq library construction and sequencing followed a previous study [71]. The genome assembly and annotation of *P. alba × P. glandulosa* [68] was used for gene alignment and annotation. Differentially expressed genes (DEGs) between *miR319a-MIMIC* and *miR319a-OE* transgenic plants and WT were identified under *P*-value <0.05. The sequencing data of this study have been deposited in the National Center for Biotechnology Information Sequence Read Archive database under accession number PRJNA1067826.

For RT-qPCR, 1 microgram of total RNA was revserse-transcribed to first strand cDNA using HiFiScript gDNA Removal RT MasterMix CWBIO CW2020M (Jiangsu, China), and UltraSYBR Mixture CWBIO (Jiangsu, China) was used for RT-qPCR analysis. 2^-△△Ct(average)^ method was used to calculate the relative expression levels genes [70]. The internal gene *18S* of poplar was used for RNA normalization as the reference gene [72]. The primers used are listed in Table S3 (see online supplementary material).

### Statistical analysis

In the experiments, data presentation patterns and experimental replicates had been shown in the corresponding figures legends. Statistical significances were determined by one-way analysis of variance (ANOVA) followed by Duncan's multiple comparison and least significant different (LSD) test using SPSS statistics. Different letters indicate significant difference among different lines at *P* < 0.05. All bar charts were represented through GraphPad Prism 8.0.2.

Acknowledgments

This work was supported by the National Key Research and Development Program of China (2021YFD2200900 to X.J.), Fundamental Research Funds of SKLTGB (CAF) (TGBFRF202301 to X.J.). We thank Prof. Dayong Li (Beijing Academy of Agriculture and Forestry Sciences) and Prof. Wanjun Zhang (China Agricultural University) for sharing plasmids pZH01: OsamiR319a-OE and miR319a-MIMIC. We thank Prof. Jinshan Gui (Zhejiang Agriculture and Forestry University) for sharing plasmids pCAMBIA2300:PdXCP1p-35Smini-GUS and pCAMBIA2300:PdDUF579-9p-35Smini-GUS. We thank Shuai Liu and Dandan Yin (State Key Laboratory of Tree Genetics and Breeding, Chinese Academy of Forestry, Beijing) for their technical assistance for ICP-MS and SEM analyses.

## Supplementary Material

Web_Material_uhae157

## Data Availability

The sequencing data of this study have been deposited in the National Center for Biotechnology Information Sequence Read Archive database under accession number PRJNA1067826.
