# Supplementary material for: MiR319a-mediated salt stress response in poplar
Source: Hortic Res. 2024 Jun 7;11(8):uhae157. doi: 10.1093/hr/uhae157 (PMC11298623; doi:10.1093/hr/uhae157)
Supplement: Web_Material_uhae157 [file web_material_uhae157.zip › Figure S1.pdf]

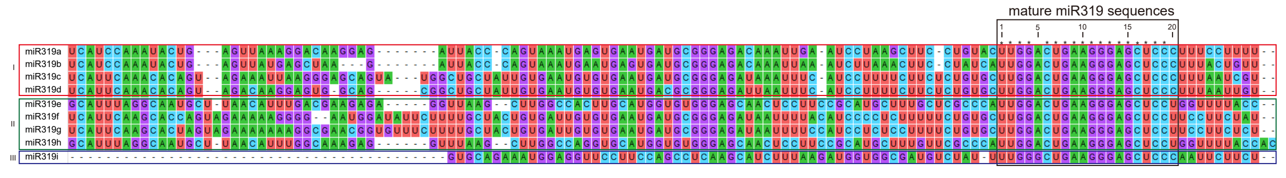
mature miR319 sequences
1
5
10
15
20
I
II
III
miR319a
miR319b
miR319c
miR319d
miR319e
miR319f
miR319g
miR319h
miR319i
