# Supplementary material for: MiR319a-mediated salt stress response in poplar
Source: Hortic Res. 2024 Jun 7;11(8):uhae157. doi: 10.1093/hr/uhae157 (PMC11298623; doi:10.1093/hr/uhae157)
Supplement: Web_Material_uhae157 [file web_material_uhae157.zip › Supplementary Figure-0514.docx]

**
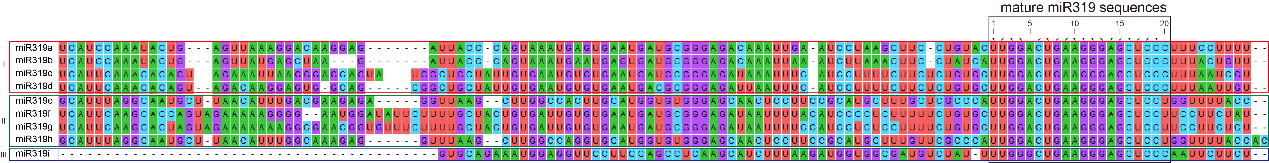
**

**Figure S1. The sequence of pre-miR319a-i and mature miR319a-i in poplar.**

**
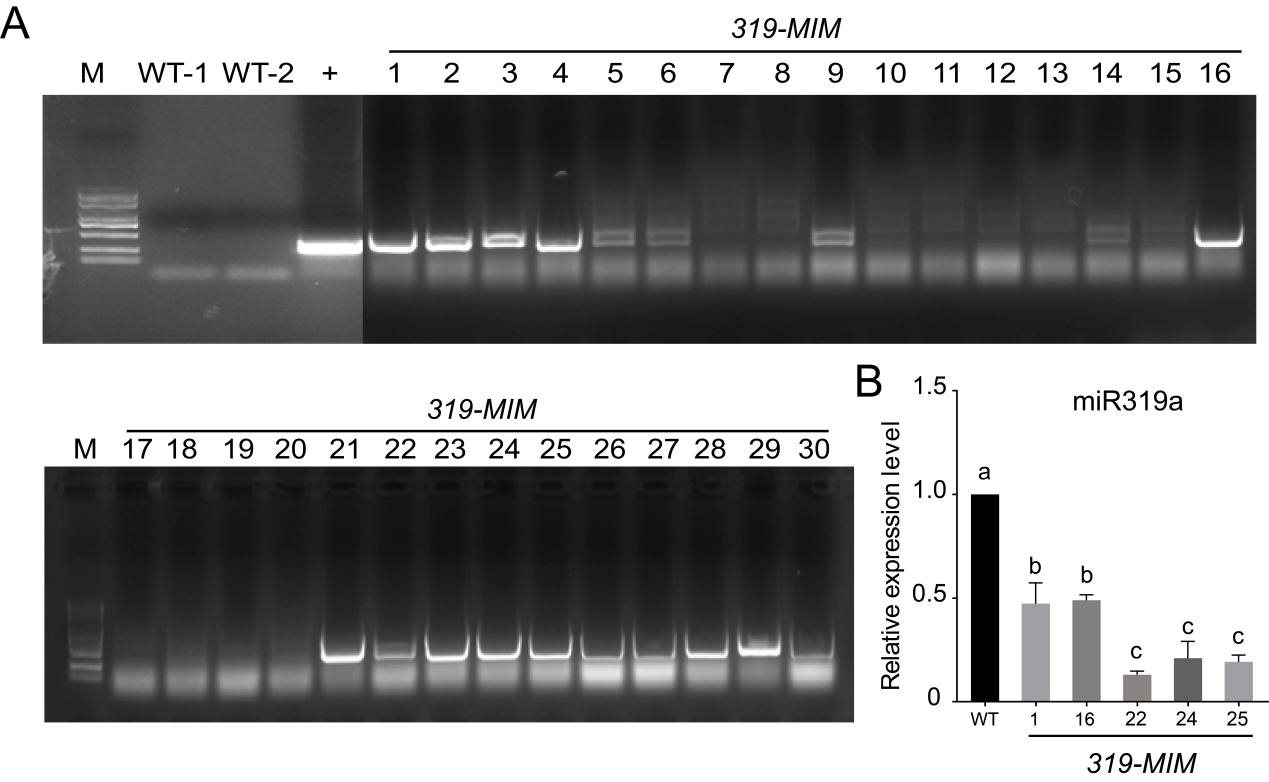
**

**Figure S2. Generation and identification of *miR319a-MIMIC* transgenic plants.**

(**A**) PCR characterization of the *miR319a-MIMC* transgenic plants. WT, wildtype. “+” represents the positive control (*miR319a-MIMIC* plasmid). (**B**) RT-qPCR detection of the abundance of the *miR319a* in the differentiating xylem of WT and *miR319a-MIMC* transgenic plants. The statistical analyses of the relative expression level are shown (means ± SD, one-way analysis of variance (ANOVA), significant differences (*P* <0.05) are indicated by different lowercase letters).


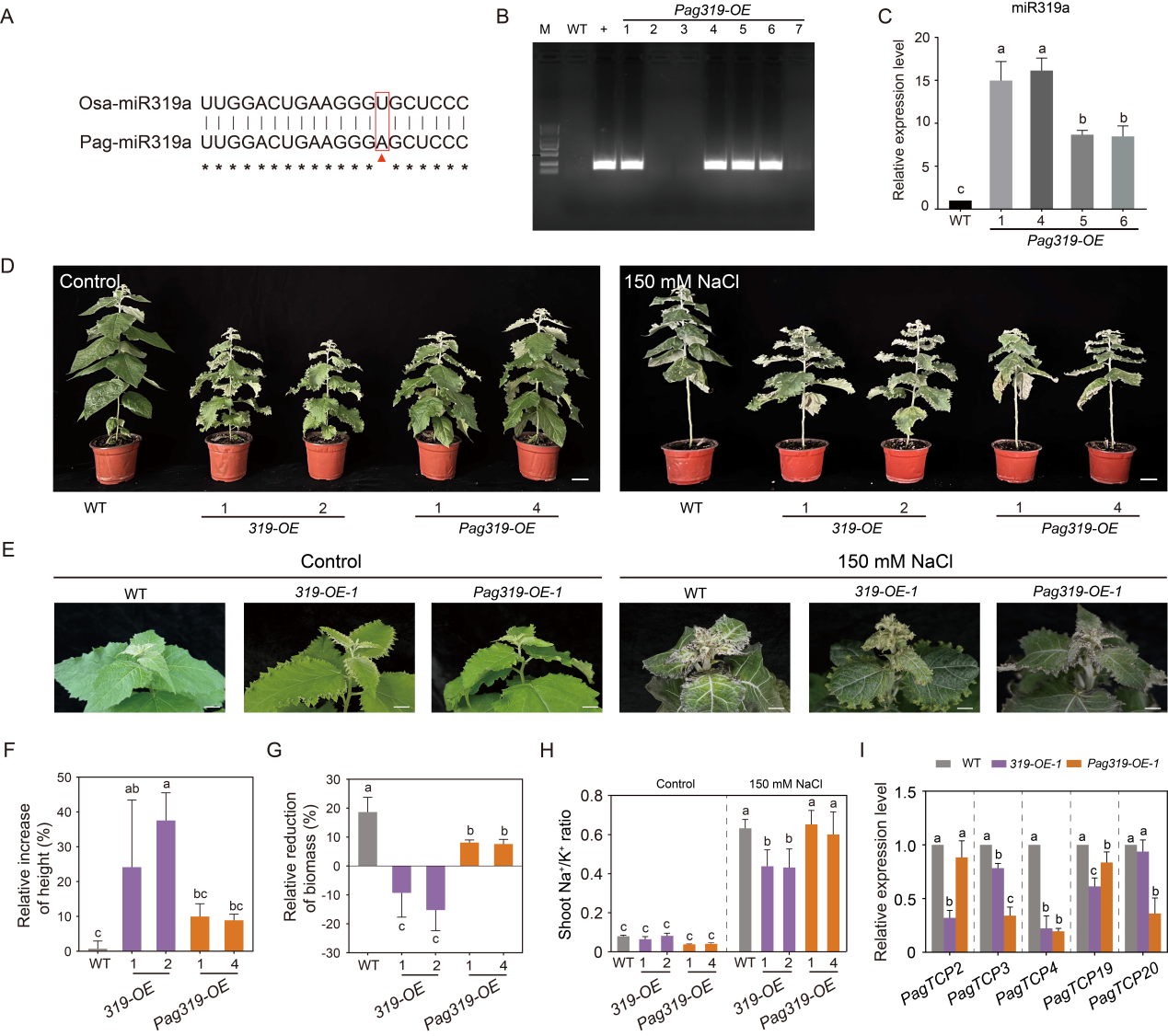


**Figure S3. Phenotypic analysis of salt tolerance in *miR319a-OE* and *PagmiR319a-OE* transgenic plants.**

(**A**) Alignment analysis of the sequences in Osa-miR319a and Pag-miR319a. (**B**) PCR characterization of *PagmiR319-OE* transgenic plants. WT, wildtype. “+” represents the positive control (*miR319a-MIMIC* plasmid). (**C**) RT-qPCR dectetion of the abundance of *miR319a* in the differentiating xylem of WT and *PagmiR319a-OE* transgenic plants. (**D**) Under control and 150 mM NaCl salt treatment, appearance of 2-months-old WT, *miR319-OE* (*319-OE-1* and *-2*) and *PagmiR319-OE* (*Pag319-OE-1* and *-4*) transgenic plants. Bar =5 cm. (**E**) Under control and 150 mM NaCl salt treatment, top leaf phenotype of WT, *miR319-OE-1* (*319-OE-1*) and *PagmiR319-OE-1* (*Pag319-OE-1*) transgenic plants. Bar =2 cm. (**F**) Relative increase of height (%) in *miR319-OE* (*319-OE-1* and *-2*) and *PagmiR319-OE* (*Pag319-OE-1* and *-4*) transgenic plants after 150 mM NaCl salt treatment. (**G**) Relative reduction of biomass (%) in *miR319-OE* (*319-OE-1* and *-2*) and *PagmiR319-OE* (*Pag319-OE-1* and *-4*) transgenic plants after 150 mM NaCl salt treatment. (**H**) Na^+^/K^+^ ratio of shoot in WT, *miR319-OE* (*319-OE-1* and *-2*) and *PagmiR319-OE* (*Pag319-OE-1* and *-4*) transgenic plants under control and 150 mM NaCl. **(I)** RT-qPCR analysis of expression levels of *PagTCP2*, *3, 4, 19* and *20* in the differentiating xylem of WT, *319-OE-1* and *Pag319-OE-1* transgenic plants.The statistical analyses are shown (means ± SD, one-way analysis of variance (ANOVA), significant differences (*P* <0.05) are indicated by different lowercase letters) in **C** and **F**-**I**.


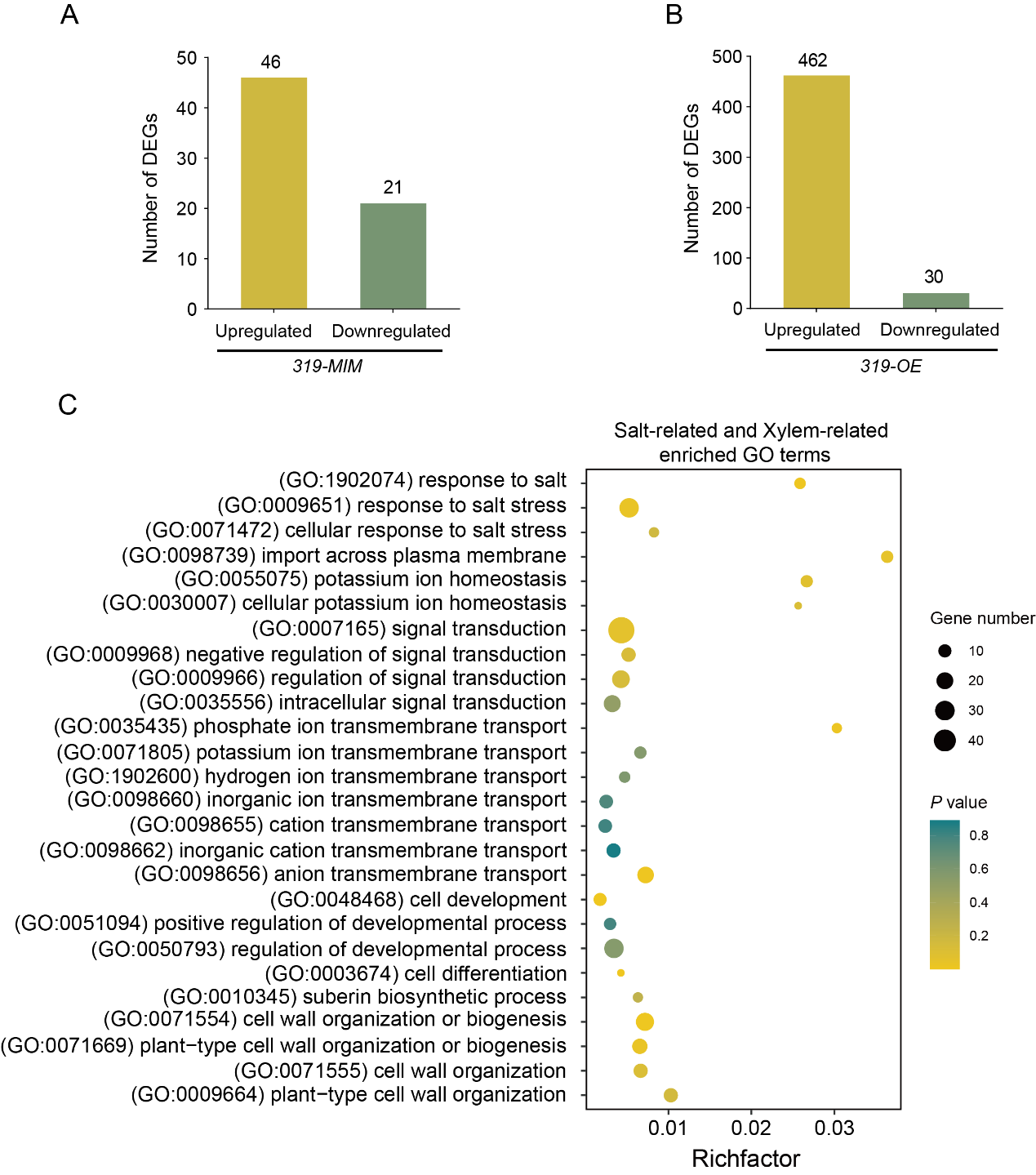


**Figure S4. Screening of differentially expressed genes (DEGs) in *miR319a-MIMIC* and *miR319a-OE* transgenic plants.**

(**A**) Numbers of DEGs in *miR319a-MIMIC* transgenic plants. (**B**) Numbers of DEGs in *miR319a-OE* transgenic plants. (**C**) GO analysis of DEGs in *miR319a-MIMIC* and *miR319a-OE* transgenic plants. The size and color of the point represent the number of genes and *P*-value (-log_10_) of the enriched GO term.


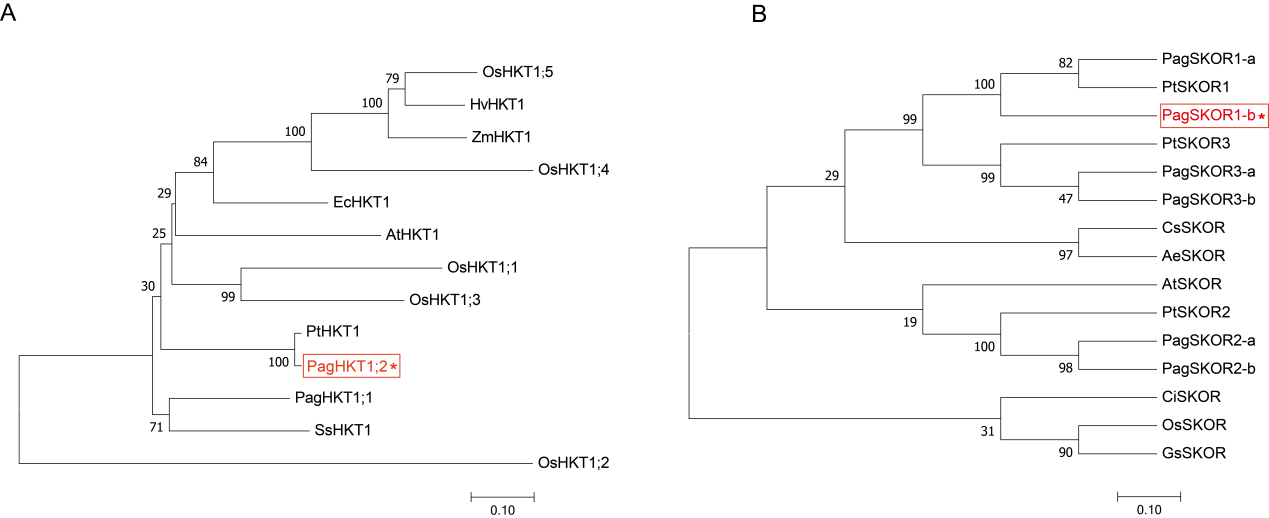


**Figure S5. Phylogenetic tree constructed with the protein sequences of PagHKT1 (A) and PagSKOR (B).**

A neighbor-joining (NJ) method in MEGA7.0 were used to generate the tree. The red highlighted genes were tested in our experiment. Abbreviations: Pag, *Populus alba* × *Populus glandulosa*; Pt, *Populus trichocarpa*; Cs, *Camellia sinensis*; Ae, *Actinidia eriantha*; Ss, *Suaeda salsa*; Ci, *Carya illinoinensis*; Gs, *Glycine soja*; At, *Arabidopsis thaliana*; Os, *Oryza sativa*; Zm, *Zea mays* L; Ec, *Eucalyptus camaldulensis*; Hv, *Hordeum vulgare*.


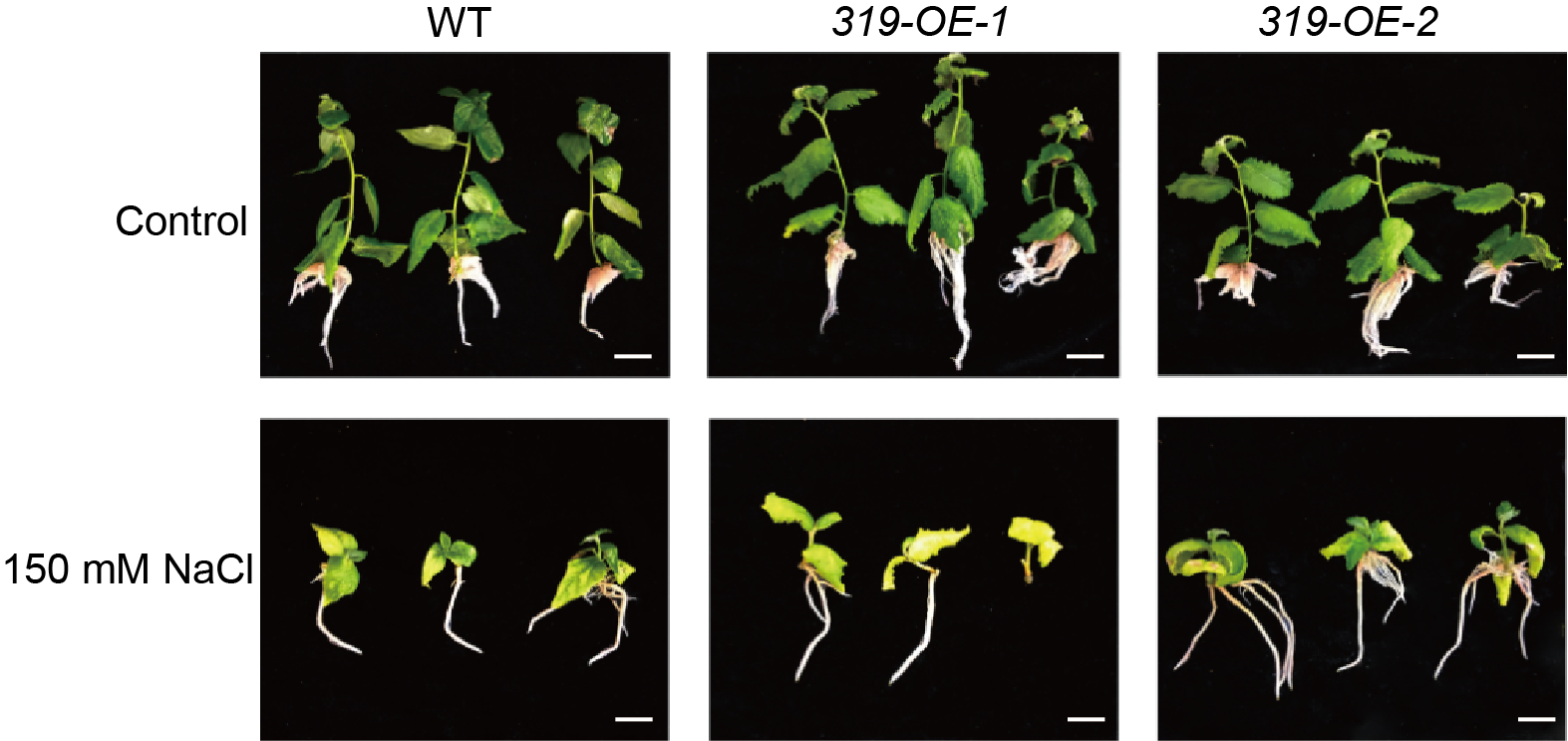


**Figure S6. Under control and 150 mM NaCl salt treatment, the appearance of tissue culture seedlings of WT, *miR319a-OE* (*319-OE-1* and *-2*) transgenic plants.** Bar = 6 cm.


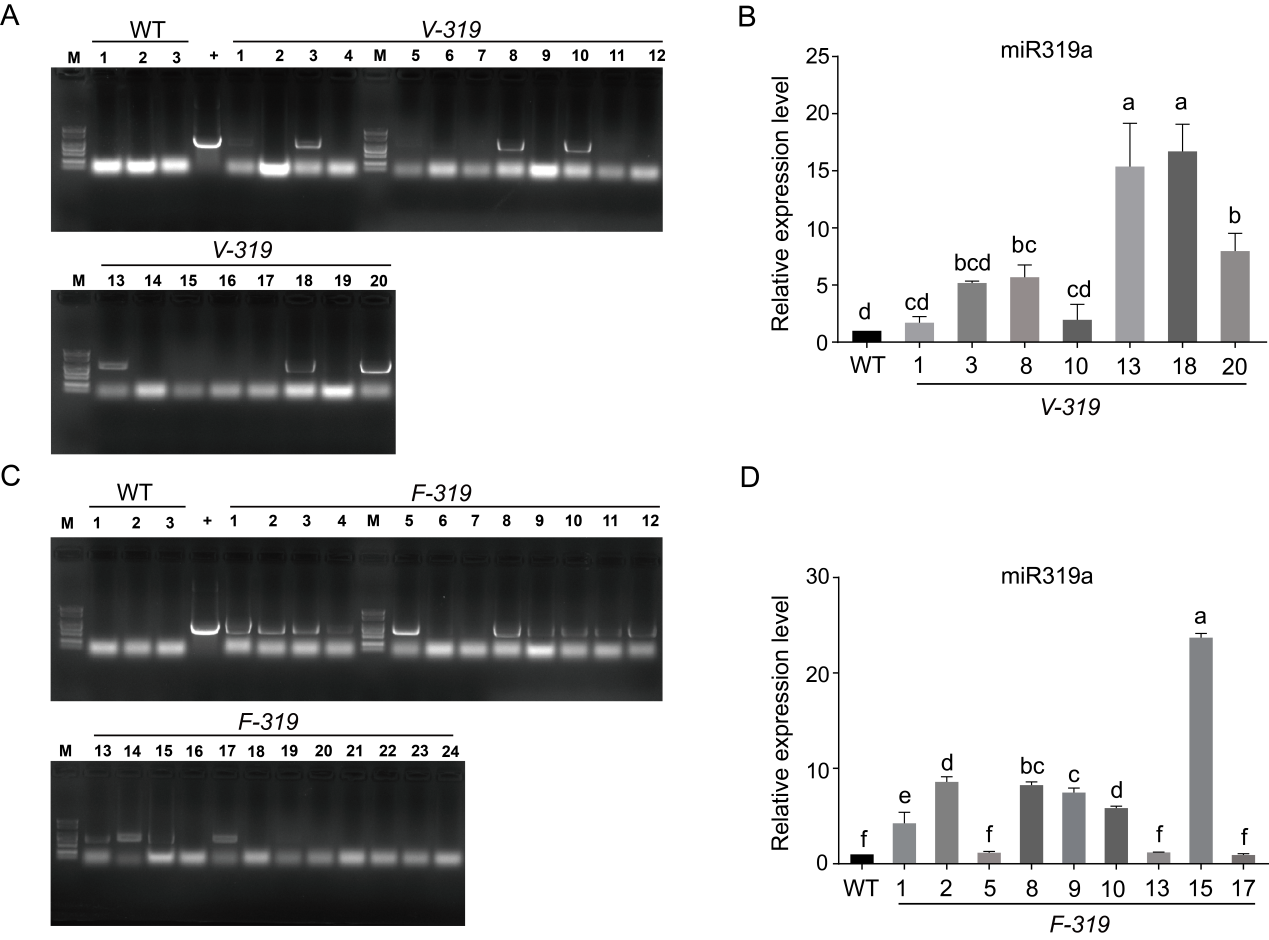


**Figure S7.** **Generation and identification of the *V-miR319a* and *F-miR319a* transgenic plants.**

(**A**) PCR characterization of the *V-miR319a* transgenic plants. WT, wildtype. “+” represents the positive control (*PdXCP1p-35Smini-miR319a* plasmid). (**B**) RT-qPCR analysis of the abundance of *miR319a* in the differentiating xylem of WT and *V-miR319a* transgenic plants. (**C**) PCR characterization of the *F-miR319a* transgenic plants. “+” represents the positive control (*PdDUF579-9p-35Smini-miR319a* plasmid) (**D**) RT-qPCR analysis of the abundance of miR319a in the differentiating xylem of WT and *F-miR319a* transgenic plants. The statistical analyses of the relative expression level are shown (means ± SD, one-way analysis of variance (ANOVA), significant differences (*P* <0.05) are indicated by different lowercase letters) in **B** and **D**.

**
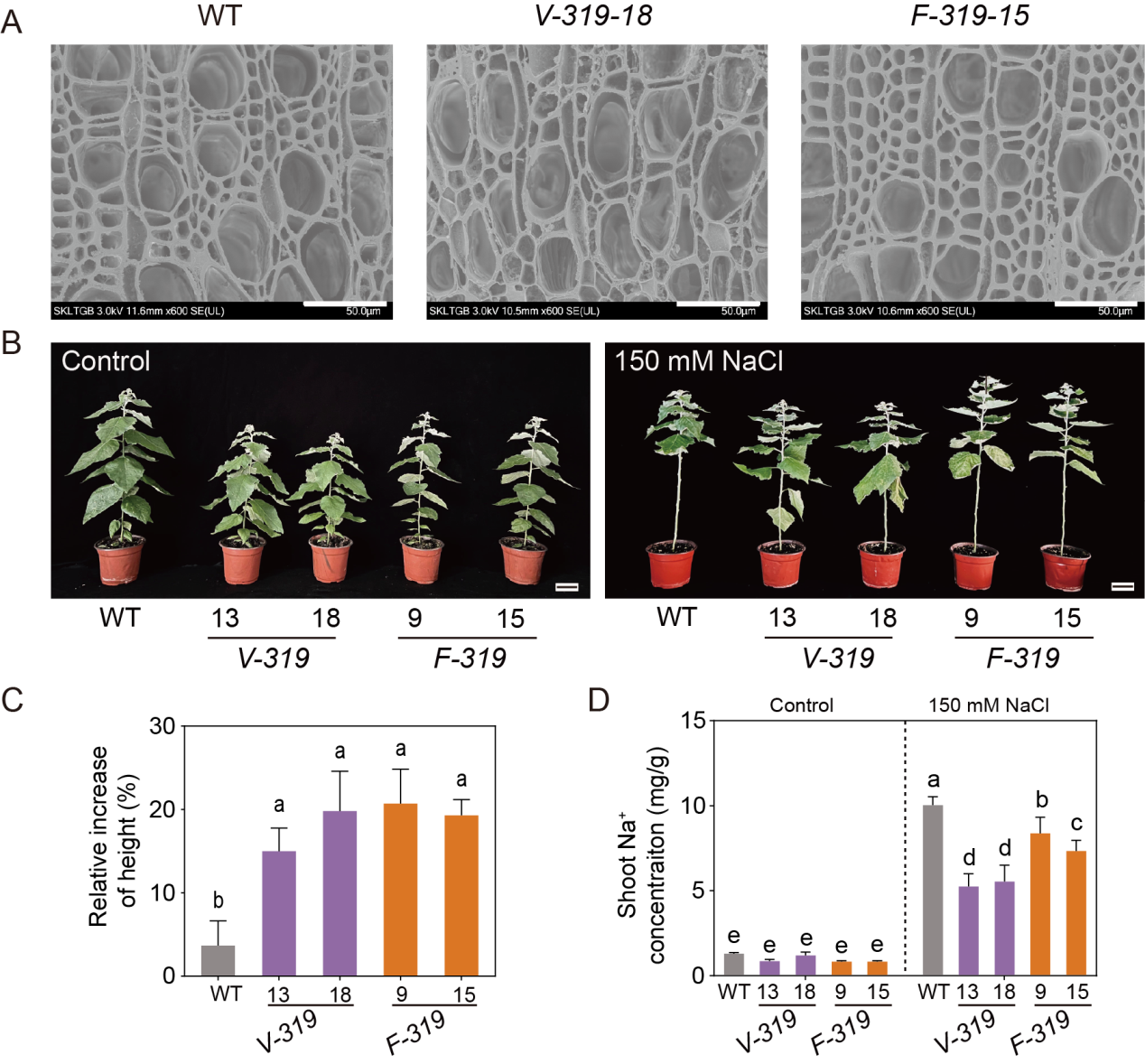
**

**Figure S8.** **Phenotypic analysis of secondary xylem and salt tolerance of *V-miR319a* and *F-miR319a* transgenic poplars.**

(**A**) Scanning electron microscopy (SEM) images of stem cross-sections of the 10th internode in WT, *V-miR319a* (*V-319-18*) and *F-miR319a* (*F-319-15*) transgenic plants. WT, wildtype. Bar =50 μm. (**B**) Under control and 150 mM NaCl salt treatment, appearance of 2-month-old WT, *V-miR319a* (*V-319-13* and *-18*) and *F-miR319a* (*F-319-9* and *-15*) transgenic plants. Bar = 5 cm. (**C**) Relative increase of height (%) in WT, *V-miR319a* (*V-319-13* and *-18*) and *F-miR319a* (*F-319-9* and *-15*) transgenic plants after 150 mM NaCl salt treatment. (**D**) Na^+^ content of shoot in WT, *V-miR319a* (*V-319-13* and *-18*) and *F-miR319a* (*F-319-9* and *-15*) transgenic plants under control and 150 mM NaCl. The statistical analyses are shown (means ± SD, one-way analysis of variance (ANOVA), significant differences (*P* <0.05) are indicated by different lowercase letters) in **C** and **D**.
