# Supplementary figures and images for: MiR319a-mediated salt stress response in poplar
Source: Hortic Res. 2024 Jun 7;11(8):uhae157. doi: 10.1093/hr/uhae157 (PMC11298623; doi:10.1093/hr/uhae157)

A

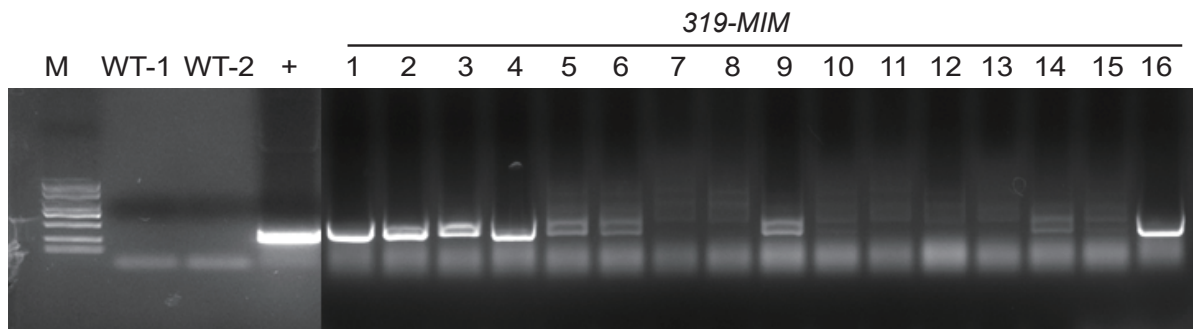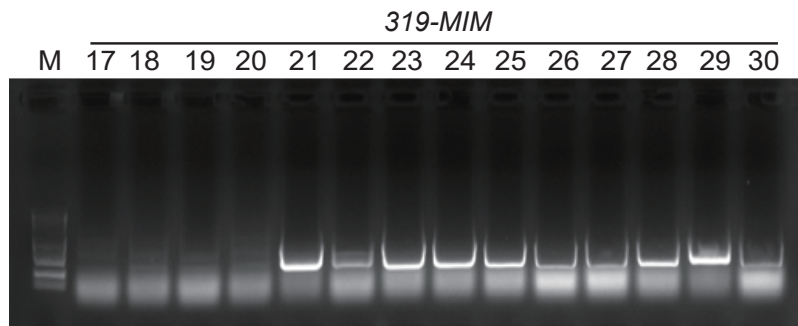

B

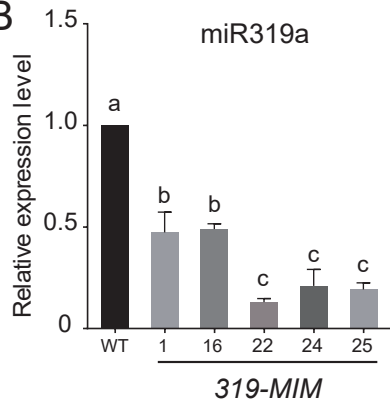

Supplement: Web_Material_uhae157 [file web_material_uhae157.zip › Figure S2.pdf]

A

Osa-miR319a UUGGACUGAAGGGUGCUC<sup>U</sup>CCC  
 |||||  
 Pag-miR319a UUGGACUGAAGGGAGCUC<sup>A</sup>CCC  
 \* \* \* \* \* \* \* \* \* \* \* \* \* \* \* \* \*

B

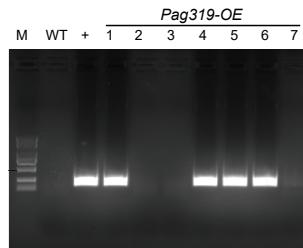

C

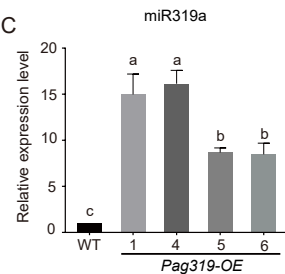

D

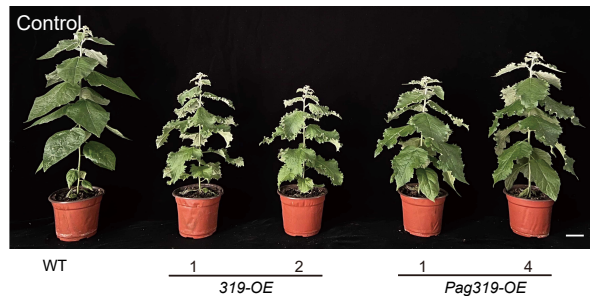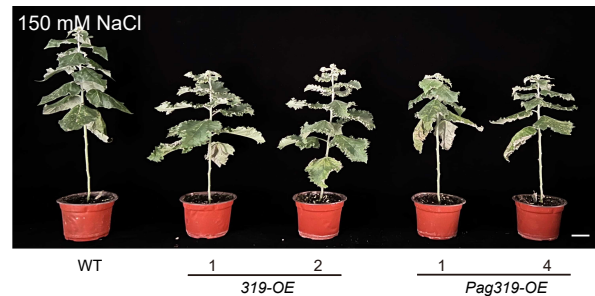

E

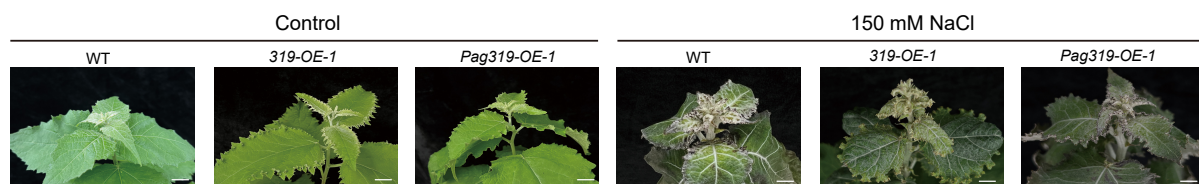

F

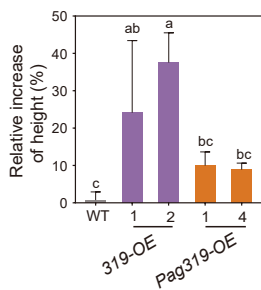

G

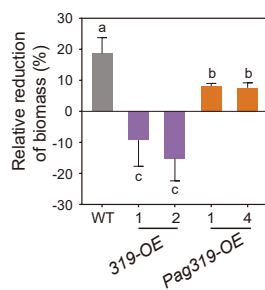

H

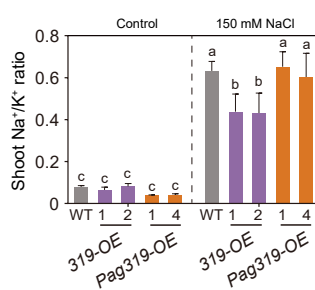

I

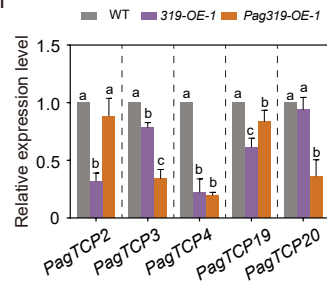

Supplement: Web_Material_uhae157 [file web_material_uhae157.zip › Figure S3.pdf]

A

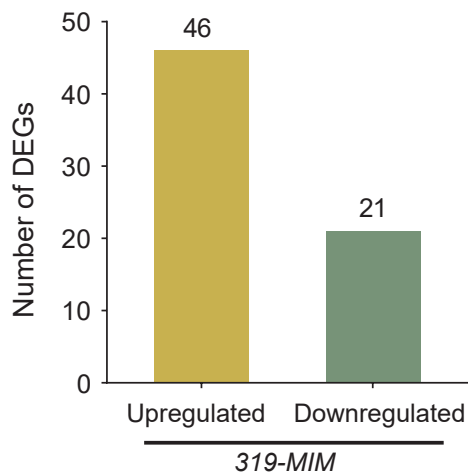

B

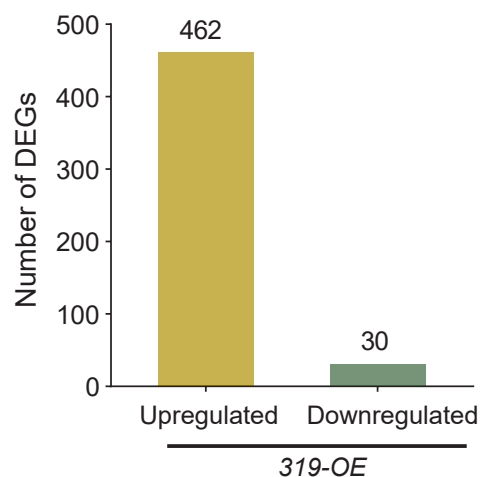

C

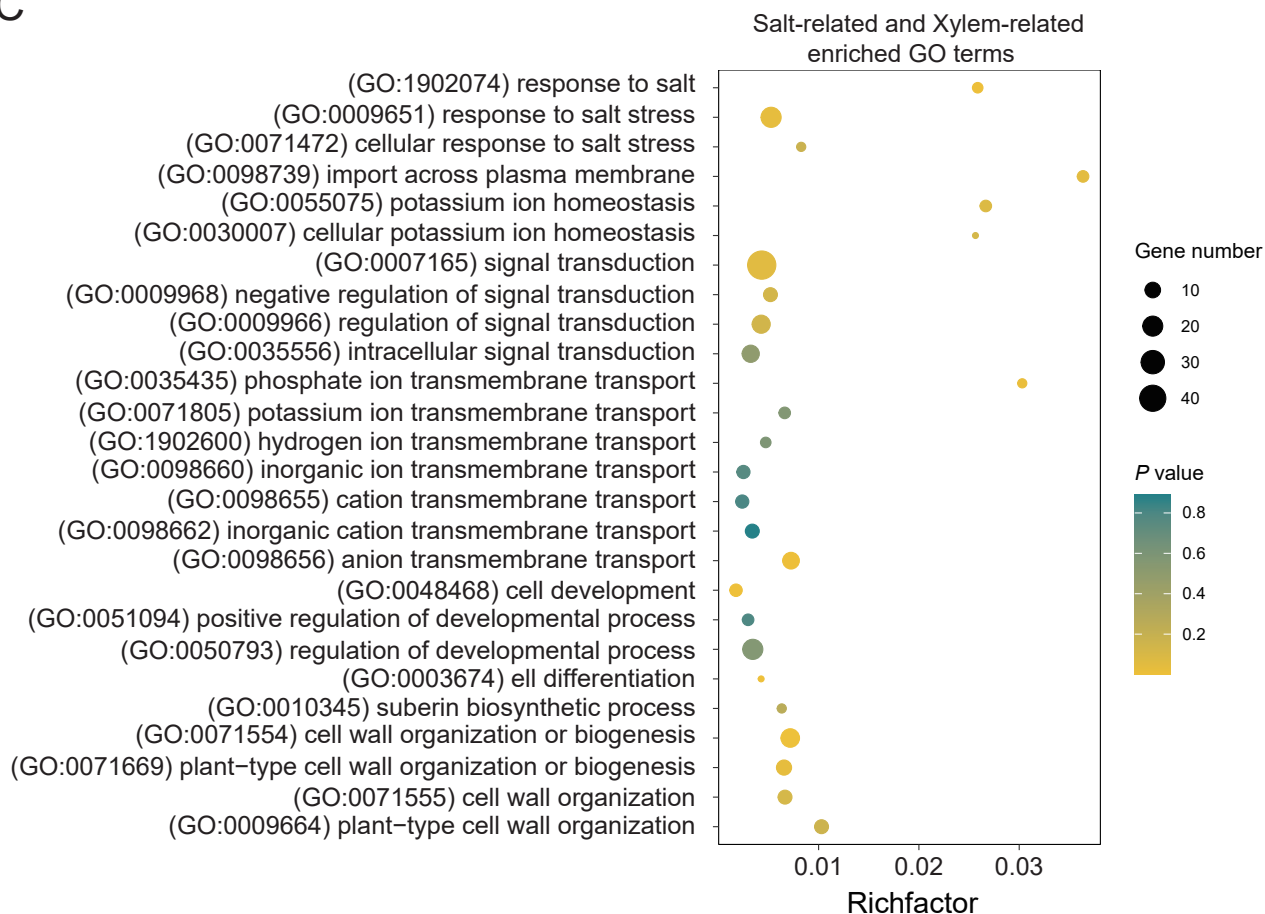

Supplement: Web_Material_uhae157 [file web_material_uhae157.zip › Figure S4.pdf]

A

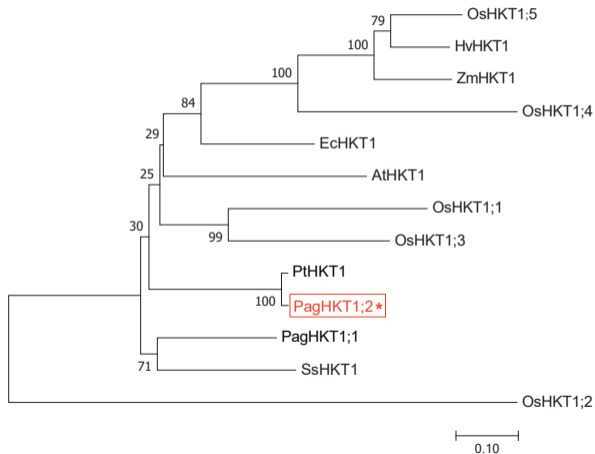

B

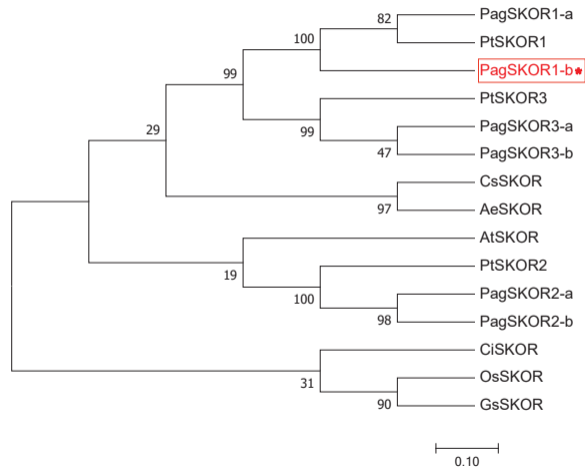

Supplement: Web_Material_uhae157 [file web_material_uhae157.zip › Figure S5.pdf]

WT

319-OE-1

319-OE-2

Control

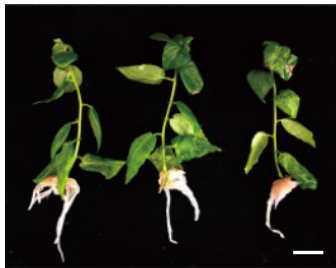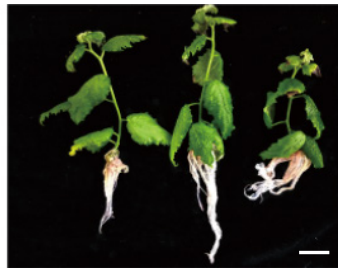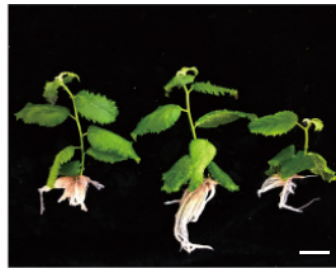

150 mM NaCl

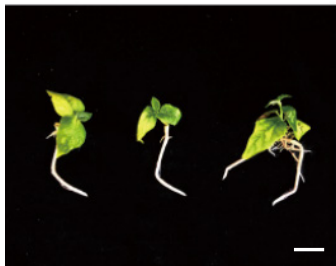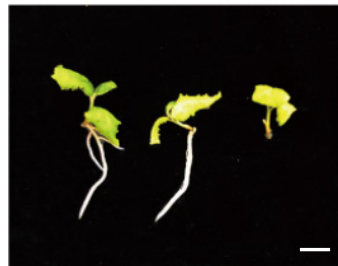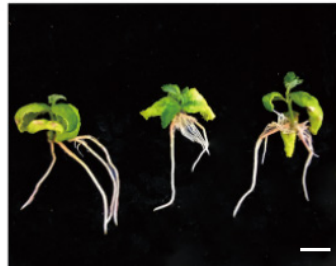

Supplement: Web_Material_uhae157 [file web_material_uhae157.zip › Figure S6.pdf]

A

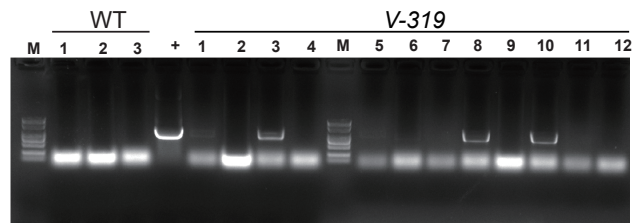

B

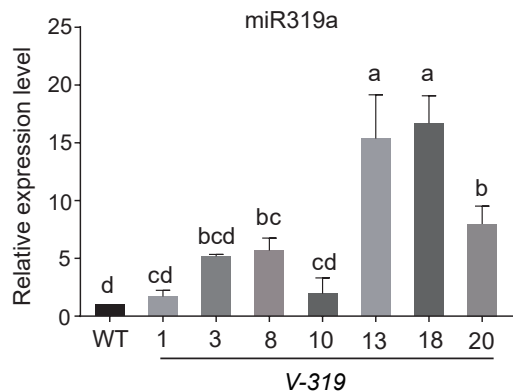

C

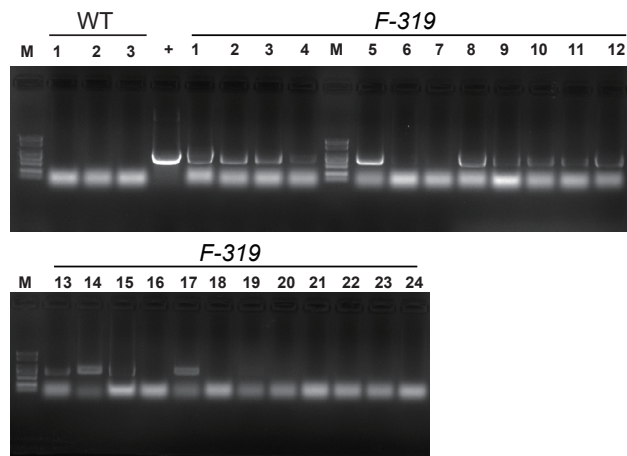

D

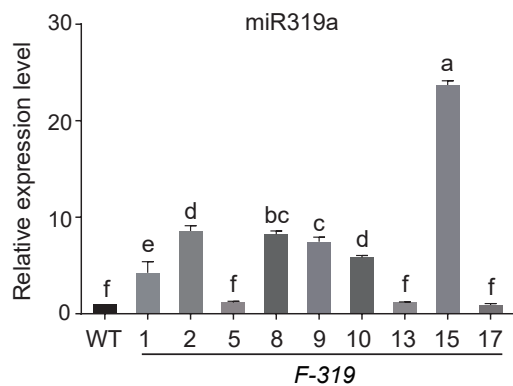

Supplement: Web_Material_uhae157 [file web_material_uhae157.zip › Figure S7.pdf]

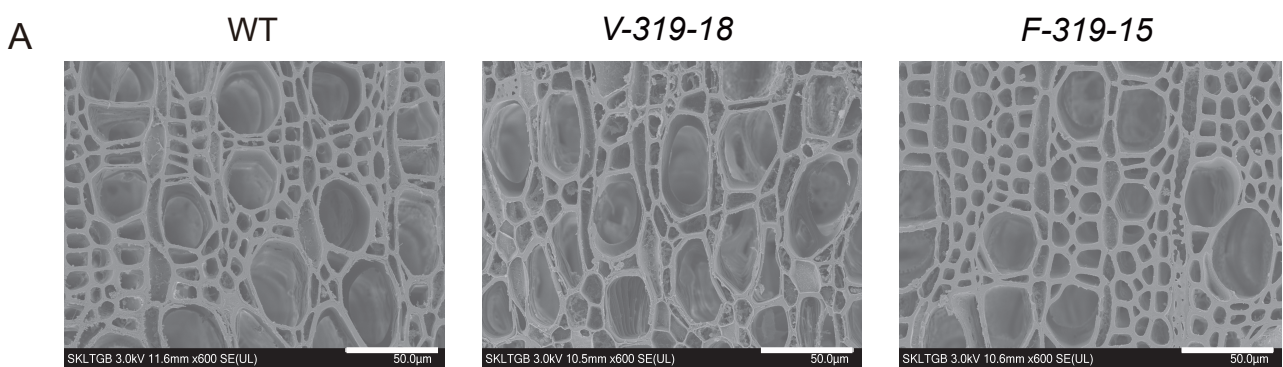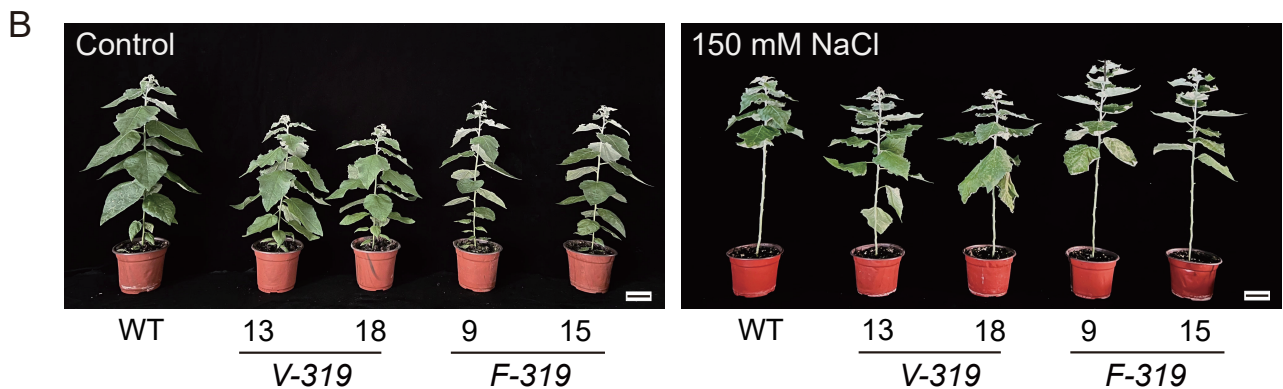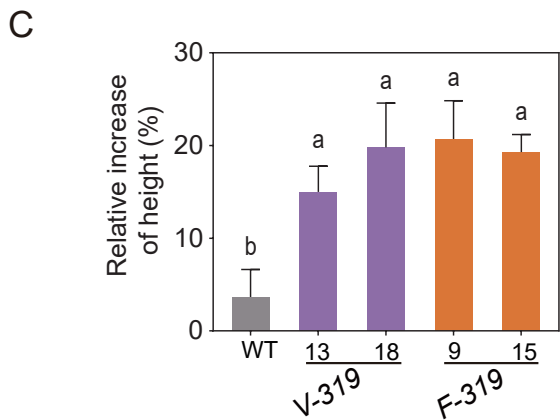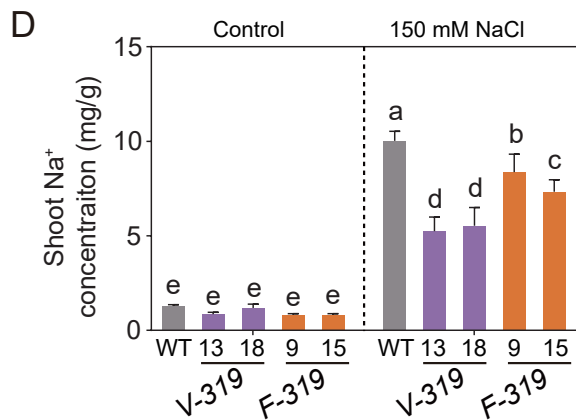

Supplement: Web_Material_uhae157 [file web_material_uhae157.zip › Figure S8.pdf]
